# Chemogenetic Activation of Excitatory Neurons Alters Hippocampal Neurotransmission in a Dose-Dependent Manner

**DOI:** 10.1523/ENEURO.0124-19.2019

**Published:** 2019-11-13

**Authors:** Sthitapranjya Pati, Sonali S. Salvi, Mamata Kallianpur, Bhupesh Vaidya, Antara Banerjee, Sudipta Maiti, James P. Clement, Vidita A. Vaidya

**Affiliations:** 1 Department of Biological Sciences, Tata Institute of Fundamental Research, Mumbai 400005, India; 2 Department of Chemical Sciences, Tata Institute of Fundamental Research, Mumbai 400005, India; 3 Neuroscience Unit, Jawaharlal Nehru Centre for Advanced Scientific Research, Bangalore 560064, India

**Keywords:** CA1, chemogenetic, CNO, DREADDs, hM3Dq, pharmacogenetic

## Abstract

Designer receptors exclusively activated by designer drugs (DREADD)-based chemogenetic tools are extensively used to manipulate neuronal activity in a cell type-specific manner. Whole-cell patch-clamp recordings indicate membrane depolarization, coupled with increased neuronal firing rate, following administration of the DREADD ligand, clozapine-N-oxide (CNO) to activate the Gq-coupled DREADD, hM3Dq. Although hM3Dq has been used to enhance neuronal firing in order to manipulate diverse behaviors, often within 30 min to 1 h after CNO administration, the physiological effects on excitatory neurotransmission remain poorly understood. We investigated the influence of CNO-mediated hM3Dq DREADD activation on distinct aspects of hippocampal excitatory neurotransmission at the Schaffer collateral-CA1 synapse in hippocampal slices derived from mice expressing hM3Dq in Ca^2+^/calmodulin-dependent protein kinase α (CamKIIα)-positive excitatory neurons. Our results indicate a clear dose-dependent effect on field EPSP (fEPSP) slope, with no change noted at the lower dose of CNO (1 μM) and a significant, long-term decline in fEPSP slope observed at higher doses (5–20 μM). Further, we noted a robust θ burst stimulus (TBS) induced long-term potentiation (LTP) in the presence of the lower CNO (1 μM) dose, which was significantly attenuated at the higher CNO (20 μM) dose. Whole-cell patch-clamp recording revealed both complex dose-dependent regulation of excitability, and spontaneous and evoked activity of CA1 pyramidal neurons in response to hM3Dq activation across CNO concentrations. Our data indicate that CNO-mediated activation of the hM3Dq DREADD results in dose-dependent regulation of excitatory hippocampal neurotransmission and highlight the importance of careful interpretation of behavioral experiments involving chemogenetic manipulation.

## Significance Statement

Designer receptors exclusively activated by designer drugs (DREADD) are tools to manipulate neuronal activity in specific circuits within the nervous system. Although these tools are routinely used to decipher the neuronal circuits responsible for a wide range of behaviors, effects of DREADD-mediated neuronal activation on excitatory neurotransmission remain poorly understood. In this study, we demonstrate using local field and whole-cell patch-clamp recordings, the complex dose-dependent effects of clozapine-N-oxide (CNO)-mediated activation of excitatory DREADD hM3Dq in hippocampal excitatory neurons on distinct aspects of neurotransmission. Our results underscore the importance of careful interpretation in the use of DREADD-mediated activation of neuronal circuits.

## Introduction

Spatiotemporal control and manipulation of neuronal activity is an essential tool to understand the causal relationship between behavior and underlying neuronal circuits. Presently, two major technologies are used to perturb neuronal function: (1) optogenetic tools which are light-activated ion channels (Britt and Bonci, 2013; Xie et al., 2013; Kim et al., 2017) and (2) chemogenetic tools which are modified G-protein-coupled receptors (GPCRs) that can be activated by a pharmacologically inert ligand clozapine-N-oxide (CNO; Pei et al., 2008; Nichols and Roth, 2009; Urban and Roth, 2015; Roth, 2016). While optogenetics is immensely useful for excitation/inhibition of neuronal activity in behavioral time scales ranging from milliseconds to several minutes, chemogenetic activation/inhibition of neuronal circuits is predominantly the method of choice for more long-term perturbations ranging from a few minutes to chronic manipulation across days (Roth, 2016; Kim et al., 2017). While both optogenetic and chemogenetic tools can be combined with inducible genetics to manipulate neuronal activity in a circuit/cell type-specific manner in precise temporal windows, optogenetics is disadvantageous for chronic manipulation due to problems such as overheating of tissue and the need to implant optical fibers by invasive surgery (Britt and Bonci, 2013; Roth, 2016; Kim et al., 2017).

The designer receptors exclusively activated by designer drugs (DREADD) hM3Dq, an engineered human muscarinic receptor, is routinely used to activate neurons (Alexander et al., 2009; Roth, 2016). The hM3Dq DREADD is coupled to Gq-signaling and like other Gq-coupled receptors, application of its agonist CNO increases accumulation of inositol monophosphate and mobilizes intracellular calcium (Armbruster et al., 2007; Guettier et al., 2009; Alexander et al., 2009; Roth, 2016). CNO administration leads to depolarization of membrane potential along with increased firing rate in hippocampal CA1 pyramidal neurons (Alexander et al., 2009), raphe serotonergic neurons (Urban et al., 2016), and arcuate nucleus AgRP neurons (Krashes et al., 2011). Although hM3Dq has been used as a tool to increase neuronal activity in several circuits including those responsible for feeding (Krashes et al., 2011; Atasoy et al., 2012), locomotion (Kozorovitskiy et al., 2012), energy expenditure (Kong et al., 2012), memory (Garner et al., 2012), and social behaviors (Peñagarikano et al., 2015), detailed neurophysiological consequences of its activation still remain poorly characterized.

Many other Gq-signaling-coupled metabotropic receptors like the metabotropic glutamate receptor 1 and 5 (mGluR1/5), muscarinic acetylcholine receptor 1 and 5 (M1/5) are known to modulate neuronal cells and circuits in distinct fashion at different doses of ligand (Volk et al., 2007; Gladding et al., 2009; Kumar, 2010; Caruana et al., 2011). Although a wide range of dosage spanning from 0.5 to 200 μM CNO has been used to record hM3Dq-mediated electrophysiological effects in acute slices (Mahler et al., 2014; Hurni et al., 2017), possible dose-dependent variation in neuronal activity has not been studied. Further, most behavioral assays are conducted from 30 min to 1 h after CNO administration (Roth, 2016), a timescale in which the physiologic effects of CNO remain poorly understood.

In the current study, we have investigated the effects of acute CNO-mediated activation of the hM3Dq receptor in the Ca^2+^/calmodulin-dependent protein kinase α (CamKIIα)-positive excitatory neurons on hippocampal neurotransmission pre and 30 min post-CNO administration. Using the Schaffer collateral pathway as the model circuit, we show that acute hM3Dq activation in excitatory neurons produces distinctly different dose-dependent effects on field currents, paired-pulse facilitation, neuronal excitability, spontaneous postsynaptic currents (sPSCs), and ionotropic glutamate receptor-mediated currents. We also observe a dose-dependent increase in intracellular calcium levels in cultured hippocampal neurons following CNO administration. Taken together, our results suggest that hM3Dq activation in hippocampal CamKIIα-positive excitatory neurons produces clear dose-dependent effects on neurotransmission, highlighting the importance of careful interpretation of behavioral studies using chemogenetic activation.

## Materials and Methods

### Animals

CamKIIα-tTA transgenic mice were received as a gift from Dr. Christopher Pittenger, Department of Psychiatry, Yale School of Medicine (Mayford et al., 1996). TetO-hM3Dq mice (Cat. No. 014093; Tg(tetO-CHRM3*)1Blr/J) were purchased from The Jackson Laboratory. CamKIIα-tTA and TetO-hM3Dq bigenic mice were bred, and the genotype of double-positive animals was confirmed using PCR-based analysis. The background strain C57Bl6/J was used for control experiments. Male mice were used for all experiments. All animals were maintained on a 12/12 h light/dark cycle and provided with ad libitum access to food and water. All animal procedures were performed in accordance with the Tata Institute of Fundamental Research animal care committee’s regulation.

### Drug administration

CNO (Tocris) was dissolved in artificial CSF (aCSF). This solution was continuously bubbled with 95% O_2_ and 5% CO_2_, and was circulated through the slice chamber during drug administration.

### Immunofluorescence and confocal imaging

As the TetO-hM3Dq mouse has a hemagglutinin (HA) tag, we performed immunofluorescence staining to visualize hM3Dq expression in the hippocampus and cortex. The CamKIIα-tTA::TetO-hM3Dq double positive or genotype-control animals (single positive for either CamKIIα-tTA or TetO-hM3Dq) were sacrificed by transcardial perfusion with saline (0.9% NaCl) followed by 4% paraformaldehyde. Coronal brain sections (40 μm) were generated using a vibrating microtome (Leica) and subjected to immunofluorescence. Following permeabilization at room temperature in PBS with 0.4% Triton X-100 (PBSTx) for 1 h, the sections were subjected to blocking solution [1% bovine serum albumin (Roche, 9048-49-1), 5% normal goat serum (Thermoscientific, PI-31873) in 0.4% PBSTx] at room temperature for 1 h. Sections were then incubated in the primary antibody, rabbit anti HA (1:250; Rockland, 600-401-384) for 4 d at 4°C which was followed by three washes with 0.4% PBSTx of 15 min each at room temperature. This was followed by incubation with the secondary antibody, goat anti-rabbit IgG conjugated to Alexa Fluor 568 (1:500; Invitrogen, A-11079) for two and a half hours at room temperature. The sections were mounted onto slides with Vectashield Antifade Mounting Medium with DAPI (Vector, H-1200). The sections were imaged using an LSM5 exciter confocal microscope (Zeiss) using identical acquisition settings for the sections from double-positive and genotype-control animals.

### Primary hippocampal neuron culture

Hippocampi were extracted from pups of CamKIIα-tTA::TetO-hM3Dq at postnatal day 0 and tail-clips were collected for genotyping. Hippocampi were dissected in HBSS-HEPES (300 mM) buffer and incubated with 0.1% trypsin-EDTA (Invitrogen) for 10 min. Neurons were dissociated in neurobasal medium supplemented with 2% B27 supplement and 0.5 mM L-glutamine (Invitrogen). Cells were plated on poly-d-lysine (Sigma) coated plates at a density of 10^6^ cells/well in neurobasal medium. Calcium imaging experiments were conducted at day *in vitro* (DIV)6–DIV8.

### Preparation of hippocampal slices

CamKIIα-tTA::TetO-hM3Dq or C57Bl/6J mice were sacrificed by cervical dislocation in accordance with the guidelines of the Jawaharlal Nehru Center for Advanced Scientific Research animal ethics committee. Following decapitation, the brain was immediately transferred to ice-cold sucrose cutting solution (189 mM sucrose, 10 mM D-glucose, 26 mM NaHCO_3_, 3 mM KCl, 10 mM MgSO_4_.7H_2_O, 1.25 mM NaH_2_PO_4_, and 0.1 mM CaCl_2_), which was bubbled continuously with 95% O_2_ and 5% CO_2_. Following dissection and removal of the cerebellum in a Petri dish containing an ice-cold cutting solution, the brain was glued onto a brain holder which was placed in a buffer tray containing ice-cold cutting solution. Subsequently, 300-μm horizontal sections were obtained using a vibrating microtome (Leica, VT-1200). The sections were then transferred to a Petri dish containing aCSF (124 mM NaCl, 3 mM KCl, 1 mM MgSO_4_.7H_2_O, 1.25 mM NaH_2_PO_4_, 10 mM D-glucose, 24 mM NaHCO_3_, and 2 mM CaCl_2_) at room temperature following which the hippocampus and the overlying cortex was gently dissected. Slices were transferred to a chamber on a nylon mesh containing aCSF bubbled with 95% O_2_ and 5% CO_2_ at 37°C. It was incubated for 30 min to 1 h to ensure stable electrophysiological responses. The slices could be maintained in a healthy state for up to 8 h and were transferred to the recording chamber as required.

### Extracellular and intracellular recording rig

The aCSF was preheated to 34°C using an online Peltier controlled temperature control system (ThermoClamp-1, Automate Scientific) and circulated through the slice recording chamber (Scientifica) at 1–2 ml min ^−1^ using a combination of peristaltic pump (BT-3001F, longer precision pump Co. Ltd.) and gravity feed. An Ag/AgCl reference wire and a thermocouple to provide feedback to the temperature control system were submerged in the recording chamber containing aCSF. All stimulating and recording electrodes were placed in the slice ∼45° to the vertical and were controlled using a micromanipulator (#1U RACK; Scientifica) which allowed movement in all three axes for correct positioning on the slice. The slice and the electrodes were visualized using an upright microscope (Slicescope pro 6000 Scientifica) with specialized optics to visualize deep tissue. The recording set up was mounted on an anti-vibration table (#63P-541; TMC) and were enclosed in a Faraday cage. The electrical noise was eliminated by grounding all electrical connections to a single ground point in the amplifier.

### Recording techniques

#### Recording electrodes

Recording electrodes were pulled from borosilicate glass capillaries (#30-0044/GC120F-10; Harvard Apparatus) using a horizontal micropipette puller (#P97, Sutter Instruments Co.). While intracellular patch electrodes (5–7 MΩ) were filled with potassium gluconate (KGlu) internal solution (130 mM KGlu, 20 mM KCl, 10 mM HEPES free acid, 0.2 mM EGTA, 0.3 mM GTP-Na salt, and 4 mM ATP-Mg salt; osmolarity adjusted to 280–310 mOsm), electrodes used for extracellular field recording (3–5 MΩ) were filled with aCSF. The microelectrodes were mounted on the electrode holder (Scientifica) so that the Ag/AgCl recording wire was in contact with the pipette solution. This holder was mounted to on a headstage which was connected to the amplifier. All signals were amplified using a Multiclamp-700B (Molecular Devices).

### Extracellular field recording

The measurement of field EPSP (fEPSP) was conducted from the CA1 stratum radiatum and was measured as the potential difference between the recording electrode and the bath electrode.

### Whole-cell patch-clamp recording

Whole-cell patch-clamp recording was conducted from somata of CA1 pyramidal neurons. A positive pressure was applied to the patch pipette filled with KGlu intracellular recording solution using a tube attached to the pipette holder. With the current and voltage offset to zero, the resistance of the electrode was checked by applying a test pulse and measuring current deflection according to Ohm’s law. The positive pressure was released when the pipette tip touched the cell surface which was confirmed both by visualization under the microscope and a change in resistance. This was followed by application of a negative pressure through gentle suction to form a tight seal, indicated by a large increase in resistance (>1 GΩ). The slow and fast capacitance were adjusted following which the patch of the membrane was ruptured by application of a gentle negative pressure and if required a strong voltage pulse. The cells with membrane potential less than –55 mV and a series resistance in the range of 5–25 MΩ were considered for future experiments.

### Stimulation protocol

The Schaffer collateral-commissural fiber pathway was stimulated using a concentric bipolar stimulating electrode (outer diameter: 125 μm, inner diameter: 25-μm platinum/iridium, CBARC75, FHC). For extracellular recordings, stimulus of a square-wave pulse of 20–100 μs in duration and 20–200 μA in amplitude was applied using an isolated direct current stimulation box (Digimeter). For all experiments, a paired-pulse stimulus was delivered at 50-ms interval per sweep with 20-s intersweep intervals and a potentiated paired-pulse was used to confirm proper placement of electrodes in Schaffer collateral. Following the acquisition of input-output (I-O) characteristic and paired-pulse ratio (PPR), a stable baseline of the fEPSP slope was established. While I-O curves were acquired at a fixed 20- to 40-μs duration and increasing stimulus intensity from 0 to 300 μA, the PPR was measured at a 10-s intersweep interval with interstimulus intervals ranging from 10–1000 ms in a quarter-logarithmic scale. CNO (1, 5, 10, or 20 μM) in aCSF was bath administered, and a constant time course was acquired for 60 min. We selected the lowest (1 μM) and highest (20 μM) CNO dosage for additional experiments. I-O response and PPR were measured before and 30 min following CNO administration, while a constant time course of stimulus-induced fEPSP was recorded. To observe the long-term effects of 20 μM CNO treatment, we also washed slices with aCSF post 30 min of CNO administration, and performed field recording for an additional 60 min. In a separate set of slices, θ burst stimulation (TBS) was used to induce long-term potentiation (LTP) following either sham (aCSF), a low dose (1 μM CNO) or a high dose (20 μM CNO) for hM3Dq DREADD activation. For the TBS-induced LTP-protocol, 13 blocks of a four-pulse 100-Hz stimuli were applied with an intersweep interval of 200 ms (5 Hz).

All measurements were done before and 30 min following CNO (1 or 20 μM) administration, except for AMPAR- and NMDAR-mediated currents for which a time course was acquired across 30 min. Intrinsic cellular properties such as resting membrane potential (RMP), input resistance (R_N_), tau, sag voltage, were calculated by a 500 ms, –100 to 180 pA, seven-step hyperpolarizing or depolarizing current injection with an inter sweep interval of 10 s. To confirm cell identity by observing the shape of the action potential and calculate the action potential threshold, a current of up to 2 nA for 2 ms was injected. to measure spiking activity, cells were held in current-clamp mode, CA1 pyramidal cell identity was confirmed qualitatively using the shape of action potential (characterized by the presence of an after-depolarization potential), and baseline was recorded for at least 5 min. Spiking was observed at both a low dose (1 μM) and a high dose (20 μM) of CNO for 2 min followed by wash in aCSF. Spontaneous currents were measured by holding the cell in voltage-clamp mode at –70 mV. To measure evoked responses, the Schaffer collateral-commissural pathway was stimulated using a concentric bipolar stimulating electrode (outer diameter: 125 μm, inner diameter: 25-μm platinum/iridium, CBARC75, FHC), and cells were voltage-clamped either at –70 mV for AMPAR-mediated currents or +40 mV for NMDAR-mediated currents.

### Calcium imaging

To detect intracellular calcium levels following administration of CNO, we used the UV-excitable calcium-sensitive ratiometric dye Indo-1 AM, Cell-permeable (I1226, Thermo Fischer Scientific). A custom-built two-photon set up with a Ti:Sapphire laser (MaiTai DeepSee, Spectra Physics) coupled to a confocal microscope (LSM 710, Carl Zeiss), as described earlier (Das et al., 2017) was used for imaging (excitation: 730–735 nm). A microscope incubator stage (Okolab) was used to maintain temperature at 37°C and 5% CO_2_ levels in the chamber. A combination of liquid copper sulfate filter (for infrared) and 400/30-nm bandpass filter was used to detect the emission of the calcium-bound dye. Primary hippocampal neurons (DIV6–DIV8) were incubated in 10 μM Indo-1 AM made in 1× HBSS (14175-079, Gibco, Life Technologies) supplemented with 100 nM CaCl_2_ for 20 min at 37°C, 5% CO_2_. Following three brief washes with 1× HBSS, a baseline fluorescence of 10 min was acquired using a 40× objective. Following this, CNO (1 or 20 μM) was bath applied, and the neurons were tracked for 30 min, with image stacks acquired every 2 min.

### Data analysis

#### Extracellular field recording data

All extracellular field recording data were analyzed off-line using Clampfit 10.5 (Molecular Devices). The slope of the rising phase of the fEPSP was used as a measure of the size of fEPSP to eliminate the possibility of contamination with population spikes. For the I-O measurements, the fEPSP slope was calculated with increasing stimulus intensity (0–300 μA). Following a paired-pulse stimulation of the Schaffer collateral pathway, a potentiation in fEPSP slope was observed. The PPR was calculated as the ratio of the fEPSP slopes following the first stimulus to the paired stimulus following interpulse intervals of 10–1000 ms on a quarter-logarithmic scale.

### Whole-cell voltage/current-clamp data

All whole-cell voltage/current-clamp data were analyzed off-line using Clampfit 10.5 (Molecular Devices). The intrinsic membrane properties were measured using voltage deflection traces from 100-pA hyperpolarizing current pulse. The R_N_ was calculated by applying Ohm’s law, R = V/I. Where V = stable state voltage and I = injected current (100 pA). The membrane time constant (τ_m_) was obtained by fitting the voltage decay with a single exponential Ae^-t/τ^ and calculating the decay constant. The sag was calculated by subtracting the steady-state voltage deflection from the peak negative-going voltage. I-O curve was obtained for the number of spikes fired in response to increasing amplitude of current injection (0–180 pA), from distinct sets of neurons. Following this, the accommodation index was calculated as the ratio of the maximum interspike interval (including the interval from the last spike to the end of the current injection) to the first interspike interval. The action potential threshold was defined as the voltage where the rate of change of voltage with respect to time (dV/dt) exceeded 10 versus^−1^. The spontaneous current amplitude and interevent intervals were calculated using an automated event detection algorithm using Mini Analysis program (Synaptosoft Inc.). An average of at least 200 events was detected from each 5-min trace before and 30 min after CNO administration and then subjected to statistical analysis.

AMPAR-mediated current was calculated as the peak amplitude of evoked current when the cell was voltage-clamped at –70 mV, and NMDAR-mediated currents were obtained as the average current 80–100 ms following the time of peak response when the cell was voltage-clamped at +40 mV. The NMDAR-mediated current decay kinetics were calculated by fitting a double exponential function in decay phase of the NMDAR-mediated current and applying the results to the following equation: τ_w_ = [I_f_/(I_f_ + I_s_)] x τ_f_ + {I_s_/(I_f_ + I_s_)} x τ_s_. Where, τ_w_ is the weighted tau, I_f_ and I_s_ are the amplitudes of fast and slow currents, τ_f_ and τ_s_ are decay constants of fast and slow currents, respectively (Rumbaugh and Vicini, 1999).

### Calcium imaging data

Calcium imaging data were analyzed using ImageJ. Following a maximum intensity projection, the time-series images were corrected for any horizontal drift using scale-invariant feature transfer (SIFT) algorithm (Lowe, 2004). A region of interest was drawn around the neurons of interest and average intensity values were calculated. Fluorescent intensities were normalized to the baseline following which statistical analysis was performed.

### Statistics

Linear regression was used to analyze the time course data, while to analyze time-binned datasets, one-way ANOVA was performed followed by Bonferroni *post hoc* analysis (GraphPad Prism, GraphPad Software Inc.). Data are expressed as mean ± SEM, and statistical significance was determined at *p* < 0.05. The spontaneous current data were analyzed using MATLAB (MathWorks). The amplitude and interevent interval were converted to corresponding cumulative probability distributions and then subjected to Kolmogorov–Smirnov two-sample comparison. Statistical significance was set at *p* < 0.001.

## Results

### Selective expression of hM3Dq DREADD in forebrain excitatory neurons

**Figure 1.**
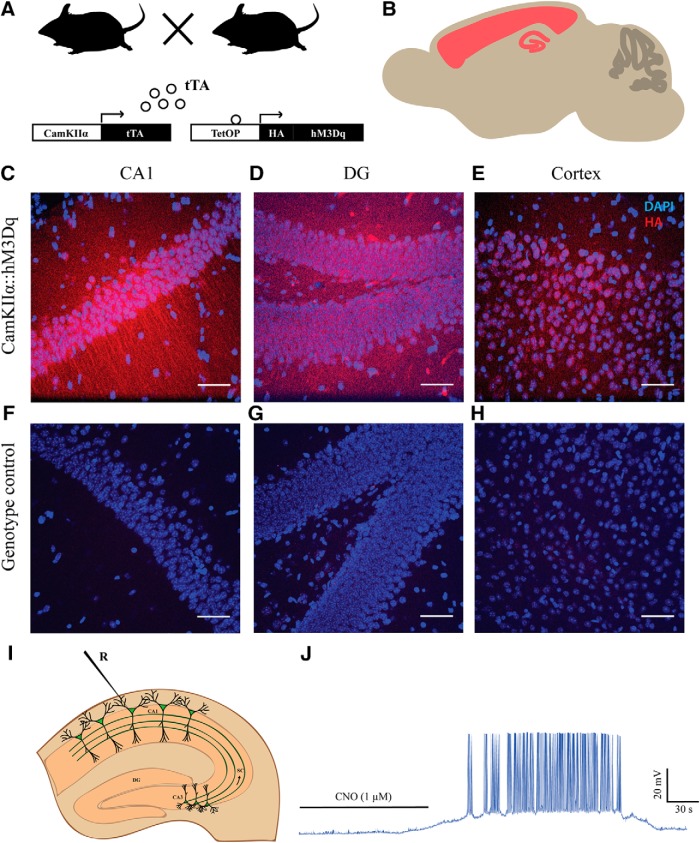
Selective expression of hM3Dq DREADD in forebrain excitatory neurons. ***A***, Shown is a schematic of experimental strategy. hM3Dq DREADD was selectively expressed in the CamKIIα-positive excitatory neurons in the forebrain. tTA: tetracycline transactivator. ***B***, Shown is a schematic of the extent of expression. Representative confocal images showing expression of hM3Dq identified by fluorescent immune-staining of HA-tag in the CA1 (***C***), DG (***D***), and cortex (***E***). No HA-tag immunoreactivity was observed in the genotype-control animals (***F–H***). Blue: DAPI; red: HA-Tag. Scale bar = 50 μm. ***I***, Shown is a schematic showing whole-cell patch-clamp recording from the somata of CA1 pyramidal cells. ***J***, Bath application of CNO (1 μM) resulted in robust spiking activity of CA1 pyramidal neurons. Refer to Extended Data Fig. 1-1 for spiking activity following bath application of 20 μM CNO. R: recording electrode.

The selective expression of hM3Dq DREADD in CamKIIα-positive excitatory neurons in the forebrain was achieved by generating a bigenic mouse created by crossing CamKIIα-tTA and TetO-hM3Dq mouse lines (Fig. 1*A*,*B*). We performed immunofluorescence analysis to detect the HA-tag and thus visualized the expression of hM3Dq in hippocampal subdivisions, including the CA1 (Fig. 1*C*) and dentate gyrus (DG; Fig. 1*D*) subfields, and also in the neocortex (Fig. 1*E*) of CamKIIα-tTA::TetO-hM3Dq animals. Analysis in single positive mice CamKIIα-tTA or TetO-hM3Dq mice indicated no HA-Tag immunofluorescence in the CA1, DG hippocampal subfields or the neocortex (Fig. 1*F–H*). Further, we confirmed the ability of CNO to induce spiking activity following bath application of both 1 μM (Fig. 1*I*,*J*, *n* = 3) and 20 μM CNO (Extended Data Fig. 1-1*A*,*B*, *n* = 3).

10.1523/ENEURO.0124-19.2019.f1-1 Extended Data Figure 1-1 ***A***, Shown is a schematic showing whole-cell patch-clamp recording from the somata of CA1 pyramidal cells. ***B***, Bath application of CNO (20 μM) resulted in spiking activity of CA1 pyramidal neurons. R: recording electrode. Download Figure 1-1, TIF file.

### Acute chemogenetic activation of hippocampal excitatory neurons differentially regulates field transmission in Schaffer collaterals in a dose-dependent manner

**Figure 2.**
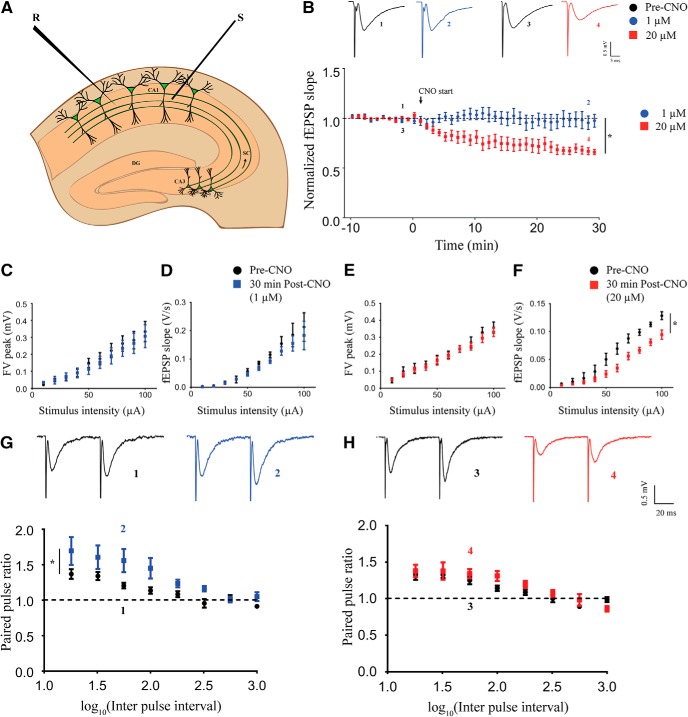
Acute chemogenetic activation of hippocampal excitatory neurons differentially regulates field transmission in Schaffer collaterals in a dose-dependent manner. ***A***, Shown is a schematic of placement of stimulating and recording electrode in the Schaffer collaterals. ***B***, Shown are representative fEPSP traces before (1) and 30 min (2) following 1 μM CNO treatment and representative fEPSP traces before (3) and 30 min (4) following 20 μM CNO treatment. Refer to Extended Data Fig 2-1 for genotype-controls and bath application of 1 μM, 5 μM, 10 μM, and 20 μM CNO. Refer to Extended Data Fig 2-2 for fEPSP time-course following CNO-washout. Bath application of CNO did not affect fEPSP time course at 1 μM, whereas it led to LTD of fEPSP at 20 μM. Stimulus intensity: 50–80 μA; 20–30 μs. The FV peak was not altered with increasing stimulus intensity either at 1 μM (***C***) or 20 μM (***E***). The fEPSP slope was not different at 1 μM (***D***), but significantly lower in response to increasing stimulus intensity at 20 μM (***F***). ***G***, Shown are representative paired-pulse-evoked fEPSP traces before (1) and 30 min (2) following 1 μM CNO treatment. A significant potentiation of PPR was observed (***G***) following 30 min of 1 μM CNO bath application. ***H***, Shown are representative paired-pulse-evoked fEPSP traces before (3) and 30 min (4) following 20 μM CNO treatment. No significant alteration in the PPR was noted following 30 min of 20 μM CNO bath application (***H***). Results are expressed as the mean ± SEM; *p < 0.05 as compared between 1 and 20 μM CNO treatment (linear regression followed by ANCOVA). S: stimulating electrode; R: recording electrode.

To investigate the effects of chemogenetic activation of CamKIIα-positive excitatory neurons on hippocampal neurotransmission, we performed time-course measurements of fEPSP slope in the stratum radiatum in response to stimulation of the Schaffer collateral-commissural fiber pathway (Fig. 2*A*). After establishing a stable baseline, we perfused aCSF containing different dosages of CNO following which fEPSP was recorded for 60 min. We did not find any significant change in the fEPSP slope over time at the lowest dose of CNO (1 μM; Extended Data Fig. 2-1*A*, *n* = 3). Interestingly, we observed a decline in fEPSP slope over time with bath application of 5 μM (Extended Data Fig. 2-1*B*, *n* = 3), 10 μM (Extended Data Fig. 2-1*C*, *n* = 4), and 20 μM (Extended Data Fig. 2-1*D*, *n* = 4) CNO. To rule out potential non-specific effects mediated by CNO, we performed the same experiment using acute hippocampal slices from the background strain, C57Bl/6J. We did not note any change in fEPSP slope over time with bath application of either 1 μM (Extended Data Fig. 2-1*E*, *n* = 3) or 20 μM (Extended Data Fig. 2-1*F*, *n* = 3) CNO. For further investigation of dose-dependent effects of CNO-mediated activation of CamKIIα-positive excitatory neurons in the hippocampus, we chose the lowest (1 μM) and the highest (20 μM) dose of CNO. As evident from these results, the CNO-mediated decline in fEPSP slope reached steady state at around 30 min after CNO administration. Therefore, we performed time-course measurements for 30 min in a separate cohort of animals. We observed no change in fEPSP time course with the low dose (1 μM) of CNO and a significant decline in fEPSP slope over time following high dose (20 μM) of CNO administration (Fig. 2*B*). The fEPSP slope time course was significantly lower at 20 μM CNO (linear regression followed by ANCOVA, *p* < 0.0001, *n* = 5 for 1 μM and *n* = 6 for 20 μM) as compared to the 1 μM CNO-treated slices. to understand whether the decline in fEPSP slope caused by administration of 20 μM CNO is transient or it can persist after the drug is removed, we washed out the CNO after 30 min and recorded fEPSP for an additional 60 min in a separate set of slices. The fEPSP slope further declined for ∼20 min following the wash out before stabilizing, and we did not observe any recovery for at least 60 min after washout (Extended Data Fig. 2-2*A*, *n* = 7). We next measured the I-O response of the pathway before CNO treatment (pre-CNO) and 30 min after CNO treatment. At low dose, we did not observe any significant difference in fiber volley (FV) peak (Fig. 2*C*, *n* = 5) and fEPSP slope (Fig. 2*D*, *n* = 5) in response to increasing stimulus intensity. At the high dose of CNO, we did not find any significant difference in the FV peak in response to increasing stimulus intensity (Fig. 2*E*, *n* = 5). Interestingly, we found that the fEPSP slope in response to increasing stimulus intensity was lower after 30 min of 20 μM CNO administration (Fig. 2*F*, linear regression followed by ANCOVA, *p* = 0.0003, *n* = 5). Paired-pulse facilitation in the Schaffer collaterals has been shown to be mediated by presynaptic mechanisms. To assess possible presynaptic effects of CNO administration, we measured paired-pulse facilitation before and 30 min following CNO administration. Strikingly, we found a potentiated paired-pulse facilitation at 30 min following 1 μM CNO administration (Fig. 2*G*, linear regression followed by ANCOVA, *p* = 0.041, *n* = 9). We did not find any significant change in PPR with increasing interpulse interval after 30 min of 20 μM CNO administration (Fig. 2*H*, *n* = 10) as compared to pre-CNO measurements.

10.1523/ENEURO.0124-19.2019.f2-1 Extended Data Figure 2-1 Bath application of hM3Dq DREADD agonist CNO had no effect on fEPSP at low dose (1 μM; ***A***). CNO treatment induced LTD at a subsequently high dosage of 5 μM (***B***), 10 μM (***C***), and 20 μM (***D***). No effect on fEPSP was observed at 1 μM (***E***) and 20 μM (***F***) CNO in slices taken from background C57Bl/6J animals. Results are expressed as the mean ± SEM. Download Figure 2-1, TIF file.

10.1523/ENEURO.0124-19.2019.f2-2 Extended Data Figure 2-2 ***A***, Bath application of 20 μM CNO for 30 min resulted in decline in fEPSP slope. The fEPSP slope continued to decrease for ∼30 min after washout with aCSF before reaching a steady-state. Recovery to baseline was not observed for at least 60 min following wash with aCSF. Download Figure 2-2, TIF file.

The above results indicate that acute chemogenetic activation of CamKIIα-positive excitatory neurons produces a long-term decline of fEPSP in the Schaffer collateral at a high dose (20 μM) of CNO, while no significant effect was observed on the fEPSP time course at the low dose (1 μM). Further, our results from the paired-pulse facilitation experiments indicate a putative potentiation of presynaptic responses in the Schaffer collaterals following chemogenetic activation of CamKIIα-positive excitatory neurons using a low dose (1 μM) of CNO.

### Dose-dependent effects of acute chemogenetic hM3Dq activation on TBS-induced LTP in hippocampal slices

**Figure 3.**
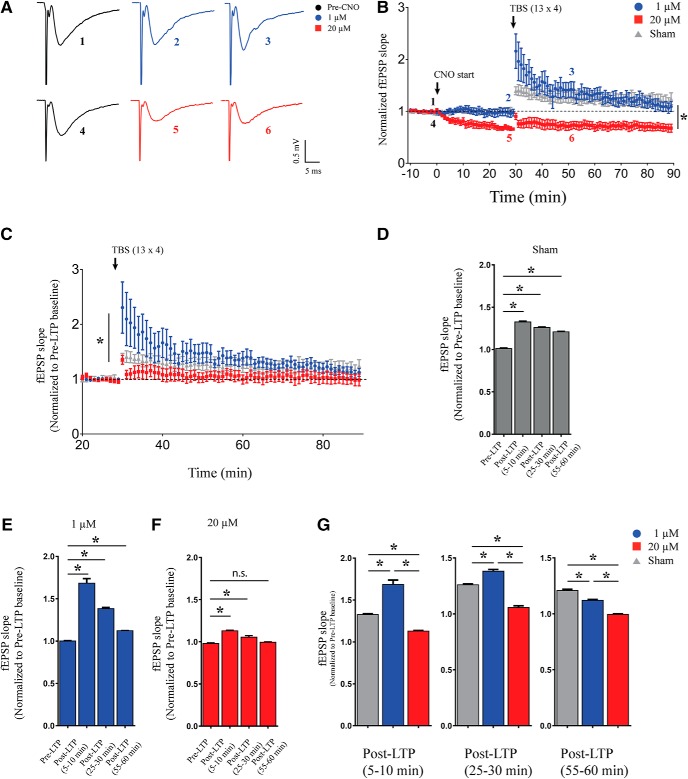
Dose-dependent effects of acute chemogenetic hM3Dq activation on TBS-induced LTP in hippocampal slices. ***A***, Shown are representative fESPS traces before, 30 min after CNO treatment and 20 min after LTP induction. ***B***, Slices with 30 min of 1 μM CNO bath application or sham-administered control, show robust induction in LTP, with failure to induce LTP at 20 μM CNO treatment. Bath application of 1 μM CNO results in a significant increase in early-phase LTP as compared to sham-treated controls. ***C***, fEPSP slope normalized to pre-LTP baseline shows robust potentiation with bath application of both sham and 1 μM CNO, but not at 20 μM CNO; *p < 0.05 as compared between 1 and 20 μM CNO treatment (linear regression followed by ANCOVA) for time-course analysis. The average fEPSP slope in 5-min time bins is significantly increased in 5- to 10-, 25- to 30-, and 55- to 60-min bins as compared to pre-LTP in aCSF bath (sham; ***D***) as well as with 1 μM CNO bath application (***E***). The average fEPSP slope in 5-min time bins was significantly increased 5- to 10- and 25- to 30-min bins as compared to pre-LTP with 20 μM CNO bath application (***F***). The average fEPSP slope was not significantly different in 55- to 60-min bin as compared to pre-LTP with 20 μM CNO bath application (***F***). Results are expressed as the mean ± SEM; *p < 0.05 as compared between pre-LTP and post-LTP (5–10 min), post-LTP (25–30 min), or post-LTP (55–60 min; one-way ANOVA followed by *post hoc* Bonferroni multiple comparison). ***G***, The average fEPSP slope in 5-min time bins is significantly increased in 5- to 10 and 25- to 30-min bins as compared to pre-LTP with 1 μM CNO bath application. The average fEPSP slope with 20 μM CNO bath application in 5- to 10- and 25- to 30-min bins was significantly lower compared to both sham-treated control and 1 μM CNO treatment. The average fEPSP slope in the 55- to 60-min bin was significantly lower with both 1 and 20 μM CNO bath application compared to sham-treated control. Results are expressed as the mean ± SEM; *p < 0.05 as compared between sham, 1 μM, and 20 μM CNO treatment for each time bin (one-way ANOVA followed by *post hoc* Bonferroni multiple comparison).

To assess whether hippocampal plasticity is modulated by hM3Dq DREADD activation at the low and high dose of CNO, we induced LTP following 30 min of either 1 or 20 μM CNO. We used a weak, physiologically relevant TBS protocol. Interestingly, we observed a robust LTP of fEPSP slope in the slices pretreated with 1 μM CNO for 30 min and also in sham-treated controls (Fig. 3*A*,*B*, *n* = 5–6/dose, *n* = 8/sham). Slices pretreated with 1 μM CNO showed supra-threshold response during early-phase of LTP, which gradually declined and showed similar levels of potentiation as compared to sham-treated controls by 30 min after LTP induction (Fig. 3*B*). However, in hippocampal slices pretreated with 20 μM CNO for 30 min, TBS failed to induce LTP (Fig. 3*A*,*B*). The slopes of LTP time course following 1 and 20 μM CNO were significantly different (Linear regression followed by ANCOVA, *p* < 0.0001, *n* = 5–6/dose). As 20 μM CNO administration for 30 min led to a decline in fEPSP slope, we re-analyzed the data by normalizing the fEPSP slope to 10 min of pre-LTP data treated as baseline which showed a significant difference between slopes of LTP time course between 1 and 20 μM CNO-treated slices (Fig. 3*C*, linear regression followed by ANCOVA, *p* < 0.027, *n* = 5–6/dose, *n* = 8/sham). For further quantification, we binned the fEPSP slope data in 5-min intervals (5-min pre-LTP, 5–10 min after LTP, 25–30 min post-LTP, and 55–60 min after LTP). Thirty minutes pretreatment with 1 μM CNO, as well as aCSF sham, led to a robust induction of LTP with potentiated fEPSP slope in 5–10, 25–30, and 55–60 min post-LTP time bins as compared to the pre-LTP time bin (*post hoc* Bonferroni multiple comparisons following one-way ANOVA, *p* < 0.05, *n* = 5 for 1 μM CNO, *n* = 8/sham; Fig. 3*D*,*E*). This analysis also revealed a small, but significant increase in fEPSP slopes in 5–10 and 25–30 min post-LTP time bins with no significant increase in the 55- to 60-min time bin as compared to the pre-LTP time bin following administration of 20 μM CNO (*post hoc* Bonferroni multiple comparisons following one-way ANOVA, *p* < 0.05, *n* = 6 for 20 μM CNO, *n* = 8/sham; Fig. 3*F*). We further compared the 5- to 10-, 25- to 30-, and 55- to 60-min post-LTP time bins between the treatment groups namely sham treatment for 30 min, 1 μM CNO for 30 min, and 20 μM CNO treatment for 30 min using one-way ANOVA. We observed a significant increase in the average fEPSP slope in the 5- to 10- and 25- to 30-min bins with 1 μM CNO bath application as compared to sham-treated controls (*post hoc* Bonferroni multiple comparisons following one-way ANOVA, *p* < 0.05, *n* = 5–6/dose, *n* = 8/sham; Fig. 3*G*). In contrast, the average fEPSP slope following 20 μM CNO bath application in the 5- to 10-, 25- to 30-, and 55- to 60-min bins significantly reduced as compared to both sham-treated control and 1 μM CNO bath application (*post hoc* Bonferroni multiple comparisons following one-way ANOVA, *p* < 0.05, *n* = 5–6/dose, *n* = 8/sham; Fig. 3*G*). In addition, the average fEPSP slope in the 55- to 60-min bin with 1 μM CNO bath application was significantly lower as compared to sham-treated controls (*post hoc* Bonferroni multiple comparisons following one-way ANOVA, *p* < 0.05, *n* = 5/dose, *n* = 8/sham; Fig. 3*G*).

These results demonstrate dose-dependent effects of CNO-mediated chemogenetic activation of CamKIIα-positive excitatory neurons on hippocampal plasticity, with slices pretreated with low dose of CNO showing robust LTP induction, whereas TBS failed to induce LTP in slices exposed to a high dose of CNO.

### Acute CNO-mediated hM3Dq DREADD activation of hippocampal excitatory neurons does not alter intrinsic membrane properties

We next performed whole-cell patch-clamp on CA1 pyramidal neurons and investigated the effects of CNO administration on intrinsic membrane properties for a duration of 30 min. First, we generated a single action potential by injecting a step current of 2 nA for 2 ms which was used to confirm CA1 pyramidal cell identity qualitatively, characterized by presence of an after-depolarization potential. We did not find any significant change in action potential threshold following 30 min of bath application of both 1 and 20 μM CNO [*n* = 24 cells (pre-CNO), 6 cells (1 μM), 11 cells (20 μM); Table 1]. As injection of a depolarizing pulse leads to firing of action potentials making a measurement of other membrane properties difficult, we next injected a hyperpolarizing step current of –100 pA for 500 ms and recorded the membrane voltage. We did not find any significant change in RMP, R_N_, sag voltage, and accommodation index following 30 min of CNO treatment (both 1 and 20 μM) as compared to the pre-CNO baseline [*n* = 24 cells (pre-CNO), 6 cells (1 μM), 11 cells (20 μM); Table 1].

**Table 1.** Acute CNO-mediated hM3Dq DREADD activation of hippocampal excitatory neurons does not alter intrinsic membrane properties

	RMP (mV)	Input resistance (MΩ)	AP threshold (mV)	τ (ms)	Sag (mV)	Sag (%)	Accomodation index
Pre-CNO	-61.31 ± 0.62	194.9 ± 10.95	-53.61 ± 0.59	26.15 ± 1.57	-5.67 ± 0.61	6.39 ± 0.61	0.45 ± 0.04
30 min Post-CNO (1 μM)	-59.24 ± 1.52	173.7 ± 17.47	-56.04 ± 2.11	23.11 ± 1.66	-4.84 ± 0.85	5.82 ± 0.84	0.36 ± 0.09
30 min Post-CNO (20 μM)	-62.30 ± 1.31	201.8 ± 13.21	-53.39 ± 1.22	27.05 ± 3.03	-4.71 ± 0.72	5.30 ± 0.74	0.47 ± 0.08

AP threshold: action potential threshold; τ: membrane time constant.

Taken together, these data indicate that chemogenetic activation of CamKIIα-positive excitatory neurons has no significant influence on intrinsic membrane properties of CA1 pyramidal neurons following 30 min of either low-dose or high-dose CNO treatment.

### Bidirectional dose-dependent modulation of excitability following 30 min of hM3Dq chemogenetic activation of hippocampal excitatory pyramidal neurons

**Figure 4.**
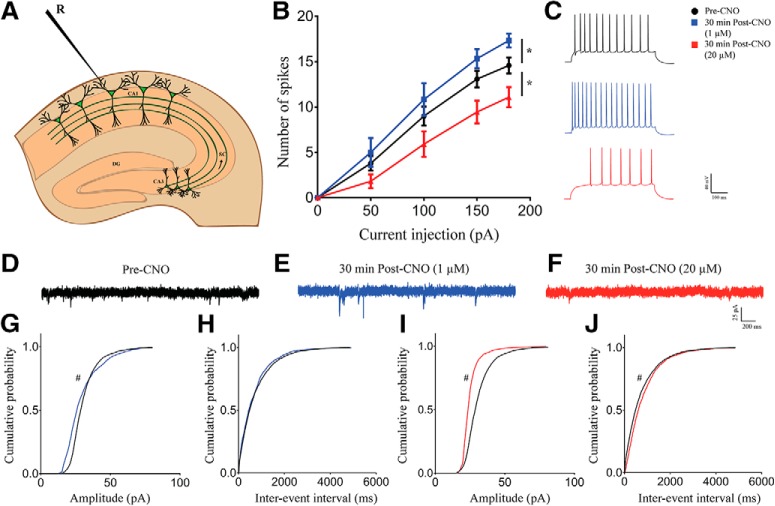
Bidirectional dose-dependent modulation of excitability following 30 min of hM3Dq chemogenetic activation of hippocampal excitatory pyramidal neurons. ***A***, Shown is a schematic depicting whole-cell patch-clamp recording from somata of CA1 pyramidal cells. ***B***, The number of spikes generated with increasing amount of current injection is enhanced with bath application of 1 μM CNO but reduced with 20 μM CNO. ***C***, Shown is a representative trace of action potentials from CA1 neurons before CNO treatment, after a 30-min bath application of 1 μM CNO, and 20 μM CNO. Results are expressed as the mean ± SEM; *p < 0.05 as compared between 1 and 20 μM CNO treatment (linear regression followed by ANCOVA). Shown are representative sPSC traces before (***D***) and 30 min after 1 μM (***E***) or 20 μM (***F***) CNO treatment. Bath application of 1 μM CNO for 30 min resulted in significantly altered sPSC amplitude with decreased cumulative probability at lower amplitude and increased cumulative probability at higher amplitude (>35 pA; ***G***), whereas no significant effect was observed on interevent interval (***H***). Bath application of 20 μM CNO for 30 min significantly decreased sPSC amplitude (***I***) and sPSC interevent interval (***J***). Results are expressed as cumulative probabilities; #*p* < 0.001 as compared to pre-CNO-treated group (Kolmogorov–Smirnov two-sample comparison). R: recording electrode.

To understand possible dose-dependent effects of CNO-mediated activation of hM3Dq DREADD in CamKIIα-positive excitatory neurons on intrinsic excitability, we injected increasing step currents and measured the number of action potentials (Fig. 4*A*). We found a significant increase in the number of action potentials produced in cells with 30 min of low dose (1 μM) CNO bath application [linear regression followed by ANCOVA, *p* = 0.04 (elevation), *n* = 24 cells (pre-CNO), *n* = 6 cells (1 μM), *n* = 11 cells (20 μM); Fig. 4*B*,*C*]. Further, there was a decrease in intrinsic excitability with 30 min of pretreatment with high dose (20 μM) of CNO, with a significant decline in number of action potentials generated by increasing amount of step current [linear regression followed by ANCOVA, *p* = 0.044 (slope), *n* = 24 cells (pre-CNO), *n* = 6 cells (1 μM), *n* = 11 cells (20 μM); Fig. 4*B*,*C*]. To understand the effects of chemogenetic hM3Dq activation on spontaneous activity of CA1 neurons, we measured sPSCs before and 30 min after activation CamKIIα-positive excitatory neurons using the low and high doses of CNO. We saw a significant difference in sPSC amplitude with 30 min of low dose (1 μM) CNO treatment with a decrease in cumulative probability at lower amplitudes (<35 pA) and increased cumulative probability at higher amplitudes [Kolmogorov–Smirnov two-sample comparison, *p* < 0.001, *n* = 13 cells (pre-CNO), *n* = 7 cells (1 μM); Fig. 4*G*]. However, we did not observe any significant change in sPSC interevent interval [*n* = 13 cells (pre-CNO), *n* = 7 cells (1 μM); Fig. 4*H*] following bath application of 1 μM CNO. Following a 30-min treatment with high dose (20 μM) of CNO, we saw a significant decrease in both sPSC amplitude [Kolmogorov–Smirnov two-sample comparison, *p* < 0.001, *n* = 13 cells (pre-CNO), *n* = 7 cells (20 μM); Fig. 4*I*] and interevent interval [Kolmogorov–Smirnov two-sample comparison, *p* < 0.001, *n* = 13 cells (pre-CNO), *n* = 7 cells (20 μM); Fig. 4*J*].

These data suggest that intrinsic excitability of CA1 pyramidal neurons is bidirectionally regulated following CNO-mediated activation of CamKIIα-positive excitatory neurons, with low dose CNO (1 μM) increasing and high dose (20 μM) of CNO treatment decreasing excitability, respectively. Further, the above data show dose-dependent bidirectional regulation of sPSC amplitude and frequency following CNO-mediated activation CamKIIα-positive excitatory neurons, with low-dose CNO increasing and high-dose CNO-mediated chemogenetic activation associated with decreased spontaneous activity.

### Dose-dependent effects of acute chemogenetic hM3Dq activation on AMPAR- and NMDAR-mediated currents

**Figure 5.**
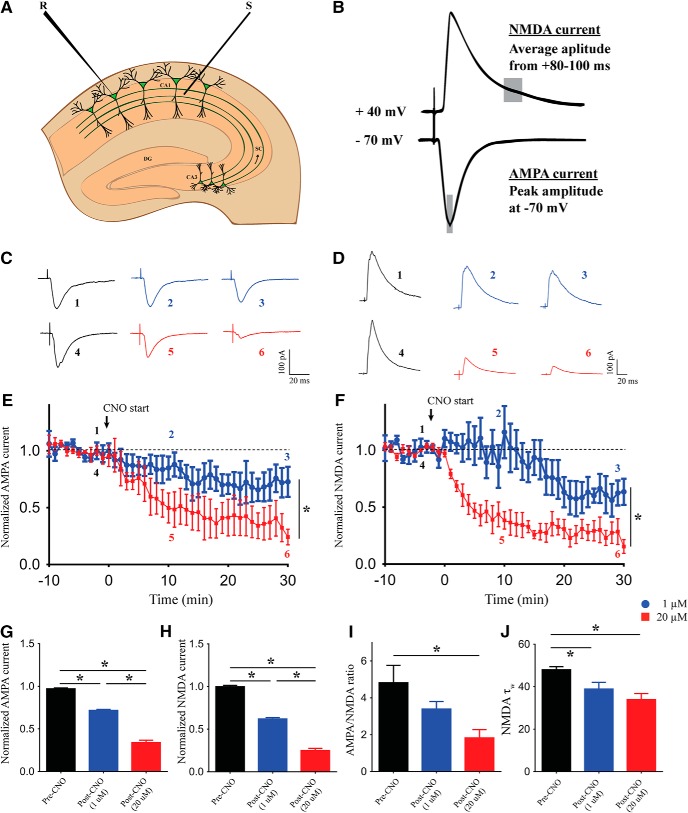
Dose-dependent effects of acute chemogenetic hM3Dq activation on AMPAR- and NMDAR-mediated currents. ***A***, Shown is a schematic depicting whole-cell patch-clamp recording from somata of CA1 pyramidal cells with stimulating electrodes places on the Schaffer collateral input pathway. ***B***, Shown is a schematic demonstrating the measurement of AMPAR- and NMDAR-mediated currents, respectively. ***C***, Shown are representative AMPAR-mediated-evoked PSC traces before and after 10 and 30 min of CNO bath application. Voltage clamped at –70 mV. ***D***, Shown are representative NMDAR-mediated-evoked PSC traces before and after 10 and 30 min of CNO bath application. Voltage clamped at +40 mV. Bath application of both 1 and 20 μM CNO results in reduced AMPAR-mediated currents, with significantly lower currents at 20 μM (***E***, ***G***). Bath application of both 1 and 20 μM CNO results in reduced NMDAR-mediated currents, with significantly lower currents at 20 μM (***F***, ***H***). AMPAR/NMDAR ratio is significantly reduced with bath application of 20 μM CNO (***I***). The time constant of NMDAR-mediated current decay (τ) is significantly decreased following 30 min of both 1 and 20 μM CNO administration (***J***). Results are expressed as the mean ± SEM; *p < 0.05 as compared between 1 and 20 μM CNO treatment (linear regression followed by ANCOVA) for time-course analysis; *p < 0.05 as compared between pre-CNO, 1 and 20 μM CNO treatment (one-way ANOVA followed by *post hoc* Bonferroni multiple comparison) for others. S: stimulating electrode; R: recording electrode.

We next sought to understand the possible cellular mechanism responsible for the decline in fEPSP and decreased synaptically-driven excitability (Fig. 2) of CA1 pyramidal cells at the higher dose of CNO treatment. As chemogenetic activation of CamKIIα-positive excitatory neurons would act by releasing glutamate and further action on glutamatergic receptors, we performed time-course measurements of evoked AMPAR- and NMDAR-mediated currents from CA1 pyramidal cells for 30 min following administration of either low (1 μM) or high dose (20 μM) of CNO (Fig. 5*A*). AMPAR-mediated current was calculated as the peak amplitude of evoked current when the cell was voltage-clamped at –70 mV, and NMDAR-mediated currents were obtained as the average current 80–100 ms following the time of peak response when the cell was voltage-clamped at +40 mV (Fig. 5*B*). AMPAR-mediated-evoked current was reduced following both 1 and 20 μM CNO administration, but the extent of decline was greater in the 20 μM CNO administered neurons as compared to 1 μM CNO administered neurons [linear regression followed by ANCOVA, *p* < 0.0001, *n* = 6 cells (1 μM), *n* = 5 cells (20 μM); Fig. 5*C*,*E*]. Similarly, we saw a reduction in NMDAR-mediated currents with both 1 and 20 μM CNO administration, and the magnitude of decline was higher in the 20 μM CNO administered neurons as compared to 1 μM CNO administered neurons [linear regression followed by ANCOVA, *p* < 0.0001, *n* = 5 cells (1 μM), *n* = 6 cells (20 μM); Fig. 5*D*,*F*]. To get a neuronal population activity, we compared average currents in 5-min bins before CNO treatment and 30 min following low and high dose of CNO treatment. We found a significant decline in both AMPAR-mediated current [one-way ANOVA followed by Bonferroni multiple comparison, *p* < 0.05, *n* = 11 cells (pre-CNO), *n* = 6 cells (1 μM), *n* = 5 cells (20 μM); Fig. 5*G*] and NMDAR-mediated current [one-way ANOVA followed by Bonferroni multiple comparison, *p* < 0.05, *n* = 11 cells (pre-CNO), *n* = 5 cells (1 μM), *n* = 6 cells (20 μM); Fig. 5*H*] at both 1 and 20 μM CNO as compared to pre-CNO currents. Further, *post hoc* Bonferroni multiple comparisons revealed that administration of 20 μM CNO significantly lowered both AMPAR-mediated current (one-way ANOVA followed by Bonferroni multiple comparison, *p* < 0.05) and NMDAR-mediated current (one-way ANOVA followed by Bonferroni multiple comparison, *p* < 0.05) as compared to 1 μM CNO treatment. Furthermore, the AMPAR/NMDAR ratio was significantly lowered with 30 min of high-dose (20 μM) CNO administration [one-way ANOVA followed by Bonferroni multiple comparison, *p* < 0.05, *n* = 8 cells (pre-CNO), *n* = 8 cells (1 μM), *n* = 7 cells (20 μM); Fig. 5*I*]. The time constant for NMDAR-mediated current decay was significantly lower following a 30-min treatment of both 1 and 20 μM CNO [one-way ANOVA followed by Bonferroni multiple comparison, *p* < 0.05, *n* = 9 cells (pre-CNO), *n* = 5 cells (1 μM), *n* = 6 cells (20 μM); Fig. 5*J*].

Taken together, these results indicate downregulation of the ionotropic glutamate receptor-mediated currents following chemogenetic hM3Dq activation of CamKIIα-positive excitatory neurons by CNO in a dose-dependent manner.

### Acute administration of CNO results in a dose-dependent increase in intracellular calcium levels in primary hippocampal neurons

**Figure 6.**
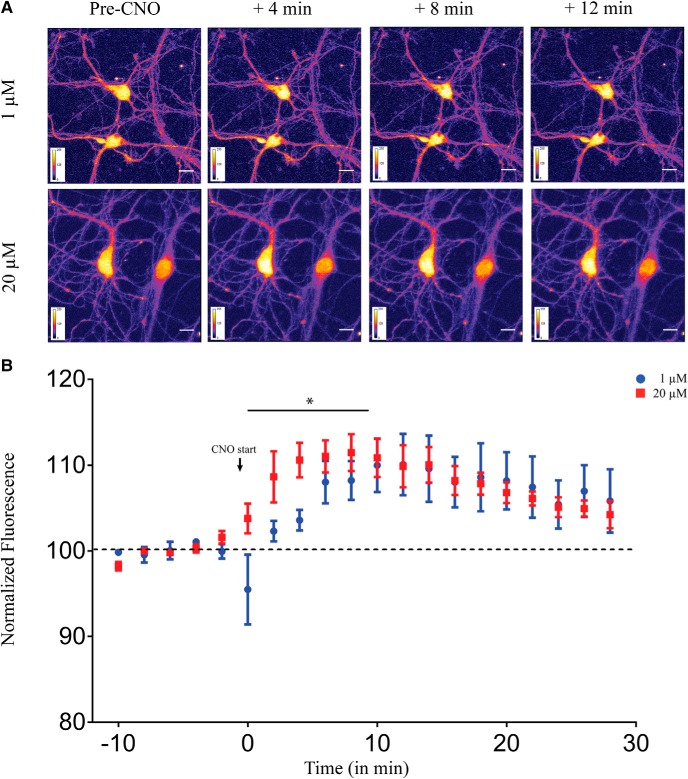
Acute administration of CNO results in a dose-dependent increase in intracellular calcium levels in primary hippocampal neurons. ***A***, Shown are representative images of primary hippocampal neurons before CNO-treatment and 4, 8, and 12 min following 1 μM (top) or 20 μM (bottom) CNO treatment. ***B***, Application of both 1 and 20 μM CNO significantly increased intracellular calcium levels as observed by increased fluorescence intensity. Treatment with 20 μM CNO resulted in a significantly faster increase in intracellular calcium levels as compared to 1 μM CNO. Results are expressed as the mean ± SEM; *p < 0.05 as compared between 1 and 20 μM CNO treatment (linear regression followed by ANCOVA).

On CNO administration the Gq-coupled hM3Dq mobilizes intracellular calcium (Armbruster et al., 2007; Roth, 2016). As calcium and its downstream signaling are key regulators of neuronal physiology, we next sought to examine whether different doses of CNO lead to differential intracellular calcium dynamics using a ratiometric calcium-sensitive dye Indo-1. Using two-photon calcium imaging, we measured intracellular calcium levels in primary hippocampal neurons following administration of either 1 or 20 μM of CNO. We observed a significant increase in calcium levels as noted from an increase in fluorescence intensity at both 1 μM (linear regression, *p* < 0.0001; Fig. 6*A*,*B*) and 20 μM (linear regression, *p* = 0.0002; Fig. 6*A*,*B*). Further, we also noted that administration of 20 μM CNO exhibited different calcium dynamics from the low dose of CNO, with a significantly faster increase in intracellular calcium levels in the first 10 min as compared to 1 μM CNO (linear regression followed by ANCOVA, *p* = 0.012, *n* = 5 cells/group, from three animals; Fig. 6*B*). Post 10 min, the intracellular calcium appeared to reach steady-state levels in cultured hippocampal neurons following acute CNO administration, and was not significantly different between 1 and 20 μM of CNO.

Taken together, our data show a dose-dependent increase in calcium levels following acute CNO administration in cultured hippocampal neurons with 20 μM CNO resulting in a significantly faster elevation in intracellular calcium levels than 1 μM CNO administration.

## Discussion

Our results provide novel insights into the dose-dependent regulation of hippocampal neurotransmission and plasticity by CNO-mediated activation of the hM3Dq DREADD in CamKIIα-positive excitatory neurons. We observed a significant decline in fEPSP slope in a dose-dependent manner with no effect at the low dose (1 μM) and the maximum decline noted at the high dose (20 μM) of CNO. Further, dose-dependent effects on hippocampal plasticity were indicated by a robust induction of TBS-evoked LTP in the presence of a low dose of CNO, which was not observed with the high CNO dose. In keeping with these results, the low dose of CNO potentiated PPR and increased excitability, accompanied by an intriguing regulation of the sPSC with enhanced probability for low amplitude events following 1 μM CNO bath application. In contrast, we observed a significant decline in excitability and sPSC at the Schaffer collateral synapses, 30 min following bath application of a high dose of CNO. Taken together, our findings provide novel evidence for the complex nature of modulation of hippocampal neurotransmission following hM3Dq DREADD activation by different doses of the ligand, CNO.

The hM3Dq DREADD is a chemogenetic tool widely used to activate neurons in a circuit and cell type-specific manner that provides critical spatiotemporal control of neurocircuitry, thus facilitating the establishment of causal links between specific neuronal pathways and behavior (Roth, 2016). Application of the DREADD ligand CNO activates the canonical Gq-signaling pathway enhancing intracellular Ca^2+^ levels (Armbruster et al., 2007; Alexander et al., 2009), and is thought to thus increase neuronal firing. Several studies have demonstrated increased firing rate and membrane depolarization in hippocampal CA1 pyramidal neurons (Alexander et al., 2009), raphe serotonergic neurons (Urban et al., 2016), and arcuate nucleus AgRP neurons (Krashes et al., 2011; Atasoy et al., 2012) in response to CNO-mediated hM3Dq activation. However, these studies employ a wide range of CNO doses ranging from 0.5-200 μM, and have performed recordings predominantly for short time scales of a few minutes following CNO administration (Mahler et al., 2014; Hurni et al., 2017). Given that most behavioral paradigms with acute hM3Dq DREADD activation operate across timescales of 30 min to several hours using diverse CNO dose ranges, it is critical to gain insight into the impact of CNO-based hM3Dq DREADD activation on neurotransmission, incorporating both different doses and longer time durations. Here, we show that CNO-mediated hM3Dq DREADD activation exerts complex, dose-dependent effects on hippocampal neurotransmission, including fEPSP slope, neuronal excitability, spontaneous and evoked activity, as well as plasticity.

Our results recapitulate aspects of prior studies examining the influence of endogenous Gq-coupled GPCR activation on hippocampal neurotransmission. Increased excitability and potentiation of an LTP phenotype are noted with the M1 muscarinic agonist, carbachol that activates Gq-signaling (Burgard and Sarvey, 1990; Bröcher et al., 1992; Natsume and Kometani, 1997). Prior evidence indicates that the mGluR1/5 agonist (R,S)-3,5-dihydroxyphenylglycine (DHPG)-dependent LTP is associated with increased excitability of CA1 pyramidal neurons and requires activation of protein kinase C, that lies downstream to Gq-signaling (Brager and Johnston, 2007). In addition, stimulation of the Gq-coupled muscarinic AChR 1 and 5 (mAChR; M1/5) leads to facilitation of LTP and synaptic transmission in both the hippocampus and visual cortex (Burgard and Sarvey, 1990; Bröcher et al., 1992; Natsume and Kometani, 1997). It is of interest that our results with the low dose of CNO (1 μM) are similar to components of the phenomena associated with activation of either the M1/5 or Group I mGluR in the hippocampus. In contrast, bath administration of both mGluR1/5 and mAChR; M1/5 agonists (Palmer et al., 1997; Cobb and Davies, 2005; Gladding et al., 2009; Kumar, 2010; Caruana et al., 2011) are also known to evoke long-term depression (LTD) at the Schaffer collateral synapses, which is similar to the effects we noted with the high dose of CNO (20 μM) used to evoke hM3Dq DREADD activation. In addition, we did not observe the recovery of the decline in fEPSP slope even after 60 min of washing with aCSF, which indicates that the effect of hM3Dq activation with high dose of the ligand evokes an LTD-like change, and could involve long-term changes in neuronal function. Previous reports demonstrate that bath application of broad-spectrum mGluR agonists (±)-1-aminocyclopentane-trans-1,3-dicarboxylic acid (ACPD; Schoepp et al., 1999), mGluR1/5 agonist DHPG (Schoepp et al., 1999), and mGluR5-specific agonist (R,S)-2-chloro-5-hydroxyphenylglycine (CHPG; Palmer et al., 1997; Fitzjohn et al., 1999; Huber et al., 2000), also induces robust LTD in several brain regions, including the Schaffer collaterals (Bellone et al., 2008; Gladding et al., 2009). It is not possible to directly compare the doses of CNO used in our study to evoke hM3Dq DREADD activation, to agonists used in prior studies to stimulate the mGluR1/5 and mAChR. However, it is of note that hM3Dq DREADD activation at distinct doses exerts effects that recapitulate aspects of prior findings with mGluR1/5 and mAChR stimulation, which are known to evoke dose-dependent, differential effects on hippocampal neurotransmission, as well as divergent effects on plasticity. Our results suggest that the engineered hM3Dq DREADD may share some similarities to endogenous Gq-coupled receptor counterparts, and thus exert complex regulation on hippocampal neurotransmission via modulation of Gq-signaling.

Our data indicate that potentially both postsynaptic and presynaptic mechanisms are triggered following bath application of CNO, and these responses likely differ across doses of CNO. We observe downregulation in AMPAR- and NMDAR-mediated currents following both low and high dose of CNO application, which supports the possibility of postsynaptic modulation by CNO-mediated hM3Dq activation. Cross talk between Gq-coupled signaling pathways and ionotropic glutamate receptors (iGluRs) has been shown extensively (Mao et al., 2005; MacDonald et al., 2007; Gladding et al., 2009). mGluR-evoked LTD is associated with downregulation of surface AMPAR (Rush et al., 2002; Huang et al., 2004; Davidkova and Carroll, 2007; Moult et al., 2008), as well as internalization of NMDA receptors from the synapse (Snyder et al., 2001). Despite the decrease in AMPAR- and NMDAR-mediated currents, we did not observe a decline in fEPSP slope following bath application of 1 μM CNO. While our results do not allow us to delineate the mechanisms responsible for this phenomenon, we could speculate a possible compensatory potentiation of presynaptic responses, supported by our observation of enhanced PPR following 1 μM CNO administration. Our current experiments do not allow us to completely reconcile the differential effects of CNO-mediated hM3Dq-DREADD activation on fEPSP, PPR, and AMPAR-mediated currents and are suggestive of possible complex underlying mechanisms that require future experiments.

We noted a potentiation of PPR and increased excitability following 30 min of CNO bath application only at the low dose. Activation of Gq-coupled receptors increases intracellular Ca^2+^ (Berridge, 1993; Nakamura et al., 2000; Larkum et al., 2003; Billups et al., 2006), it is possible that bath application of different doses of CNO could lead to different concentrations of intracellular Ca^2+^, both at the presynapse and postsynapse. Different levels and temporal kinetics of intracellular concentration of Ca^2+^ are capable of recruiting distinct signaling pathways (Berridge, 1998; Augustine et al., 2003; Thomas et al., 1996). With a body of literature showing coupling of intracellular Ca^2+^ to Gq-coupled GPCR activation (Berridge, 1993, 2009; Nakamura et al., 2000; Larkum et al., 2003; Billups et al., 2006), differential responses including iGluR currents and sPSC may arise as a consequence of differential intracellular Ca^2+^ dynamics. Further careful investigation is required to tease out the exact role of downstream Ca^2+^-mediated signaling in mediating complex regulation of neuronal physiology by hM3Dq DREADD activation.

Our data differ from the results of López et al. (2016), demonstrating enhanced LTP following bath application of CNO (5 μM) in C57Bl/6 animals virally expressing hM3Dq in CamKIIα-positive neurons in the CA1. The differences in results could be due to the fact that the expression of hM3Dq was driven using viral versus bigenic mouse lines, and was spatially restricted to just the CA1 versus the entire hippocampus, in addition to other differences in age of animals used. This further substantiates the argument to be careful when interpreting neuronal activation/behavioral data using chemogenetic tools. To rule out off-target effects of CNO and its metabolites, we performed fEPSP time-course measurements in hippocampal slices derived from the background C57Bl/6J mice lacking the hM3Dq receptor. We did not observe any significant effect both at 1 and 20 μM of CNO administration for 1 h. Although our results ruled out any off-target effects on fEPSP time course in the Schaffer collaterals, further control experiments would be useful to rule out non-specific effects on other physiologic measures.

Behavioral studies using hM3Dq activation have employed a wide range of doses of CNO ranging from 0.1 to 20 mg/kg (Alexander et al., 2009; Ray et al., 2011; Mahler et al., 2014; MacLaren et al., 2016). Recent studies in rat models indicate that intraperitoneal injection of CNO (5 mg/kg) results in plasma concentrations of up to 2 μM CNO, which persist in the micromolar range for 90 min (MacLaren et al., 2016). However, a report by Gomez et al., did not find detectable CNO levels in the brain following systemic [^3^H] CNO (∼5 μg/kg) intraperitoneal injections in C57BL/6J mice, although the dose used was very low as compared to that standardly used for behavioral experiments (Gomez et al., 2017). It is important to consider that the metabolism and pharmacodynamics of CNO could vary across strains, species, sex, and age, and our results motivate future experiments to precisely measure CNO levels within distinct brain regions and their physiologic effects across distinct doses and time scales.

In conclusion, our data elucidate that treating hM3Dq DREADD activation simply as neuronal excitation, misses the nuances of regulation of various aspects of neurotransmission over time scales and across dose ranges. It is possible that different doses of CNO can have very different pharmacological effects including recruitment of differential downstream signaling pathways, differential internalization kinetics, and cross talk with other ionotropic and metabotropic receptors. The signaling pathways recruited by activation of GPCRs like hM3Dq are arguably distinct from those recruited during the use of optogenetic methods to activate ion channels such as ChR. Although chemogenetic tools are invaluable in the quest for neuronal circuit/cell-specific modulation of behavior, our results motivate future experiments to carefully address the physiologic effects of hM3Dq DREADD activation as an important component during interpretation of behavioral data.

## Synthesis

Reviewing Editor: Thomas McHugh, RIKEN Center for Brain Science Decisions are customarily a result of the Reviewing Editor and the peer reviewers coming together and discussing their recommendations until a consensus is reached. When revisions are invited, a fact-based synthesis statement explaining their decision and outlining what is needed to prepare a revision will be listed below. The following reviewer(s) agreed to reveal their identity: Zafar Bashir, Weinan Sun.

Summary and Synthesis

The current study examines the long-term, dose-dependent effects of hM3Dq activation on Schaffer-collateral synapses and CA1 pyramidal neurons. The authors employ in vitro electrophysiology to address the previous assumption that activation of excitatory DREADDs simply increases neuronal excitability. However, the manuscript suggests that excitatory hM3Dq DREADD activation can have a multitude of effects dependent on the concentration of the ligand CNO. These findings call for caution when interpreting effects of hM3Dq activation in vivo.

While both reviewers found the manuscript interesting and of potential value to the field, they raised a number of questions that should be considered in revising the manuscript.

---------------

Major points to address:

1. The authors must clarify how the concentrations of CNO used in this study (1-20uM) relate to CNO doses used in in vivo studies. Without knowing this, the current study may be interesting but doesn't really help when planning in vivo studies. The reviewers realize it may be difficult to get direct data speaking to this point, however they should discuss known aspects of the pharmacokinetic profile of CNO after injection, and considering CNO could be metabolized quickly in vivo, how likely CNO concentration in CSF could rise to the order of 20 μM.

2. Authors should show what happens to the fEPSP amplitude when 20 μM CNO is washed out after 30-60min of continuous application. Would the amplitude recover to the previous size? This will add insights to whether CNO's effect is transient or it is altering some long-lasting cellular functions (eg. whether the effect of CNO is only desensitizing the glutamate receptors, or it is causing an effect analogous to LTD).

3. Authors showed, to the contrary of the commonly held view that hM3Dq's action is excitatory, there is an inhibitory effect of hM3Dq activation in a dose-dependent manner. The authors should also do whole-cell current clamp recordings and show that CNO at various doses can indeed induce a transient excitatory effect in their hands to make sure their system is working correctly as intended (eg. CNO can induce depolarizations and cause firing of CA1 pyramidal neurons early on).

4. Fig 2: there is no change in baseline transmission with 1uM CNO; so how is there a change in PPR? If there is a change in release probability as the authors suggest, then one might expect a decrease in baseline transmission.

5. Fig 2: The effects of 20uM CNO are a little confusing; there is a depression of baseline transmission but no effect on PPR - if the depression is due to a presynaptic effect then PPR should change. Alternatively, one might speculate that the baseline depression of fEPSPs with 20uM CNO might result from the known depolarisation effects of activation of Gq coupled receptors. However, there was no reported depolarisation effects of either concentration of CNO.

6. 1 (and 20uM) CNO lead to a decrease in AMPA EPSC amplitude; so why was there no decrease in field EPSP amplitude with 1uM CNO. In addition, given the effect of 1uM CNO on NMDA EPSCs it is surprising that LTP was still induced in the presence of 1uM CNO. The authors need to discuss why 1 μM CNO did not change the amplitude of fEPSPs, but instead reduced AMPAR and NMDAR EPSCs in whole-cell recordings.

7. Regarding the LTP experiments it was not clear what the effect of theta stimulation was in the absence of CNO, these data should be included. Next, was this a subthreshold or suprathreshold protocol for LTP under control conditions? Further, re-normalising for depression of baseline is fine, but ideally the stimulus intensity should be increased to the original baseline to control for this depression - this would be the ideal way of knowing if 20uM CNO prevents LTP per se or whether the LTP block is simply due the decrease in baseline strength.

8. Fig 5: why is there a delay in effect on NMDA EPSCs by 1uM CNO?

Activation of Gq receptors has established effects on a variety of intrinsic properties; in the initial experiments reported on P19-20 in this manuscript why are there no effects of CNO?

Some of the discussion (P26 onwards) was very speculative, especially when trying to link hM3Dq effects with mGluR and muscarinic receptor activation.

Minor points:

1.Many places throughout the text and figures (eg. Line 321), “uM” should be spelled as “μM”.

2. Please explain what the circles mean in figure 1.

## Author Response

Major points:

1. The authors must clarify how the concentrations of CNO used in this study (1-20uM) relate to CNO doses used in in vivo studies. Without knowing this, the current study may be interesting but doesn't really help when planning in vivo studies. The reviewers realize it may be difficult to get direct data speaking to this point, however they should discuss known aspects of the pharmacokinetic profile of CNO after injection, and considering CNO could be metabolized quickly in vivo, how likely CNO concentration in CSF could rise to the order of 20 μM.

For activating hM3Dq DREADDs in rodent behavioral studies, experimenters have employed a wide range of dosages of CNO ranging from 0.1 mg/ kg to 20 mg/ kg (Alexander et al., 2009; MacLaren et al., 2016; Mahler et al., 2014; Ray et al., 2011). According to a recent result, intraperitoneal injection of (5 mg/ kg) CNO results in plasma concentrations of CNO of about 2 μM which persists in the micromolar range for at least ninety minutes in male Long-Evans rats (MacLaren et al., 2016). Although another report by Gomez et al. did not find any detectable CNO within the brain following systemic [3H] CNO intraperitoneal injections in C57BL/6J mice, the dose used by them (~5 μg/ kg) was substantially lower compared to that used routinely for behavioral experiments (Gomez et al., 2017). Our unpublished observations with liquid chromatography-Mass spectrometry (LC-MS/MS) measurements suggest around 80 μM plasma CNO levels thirty minutes following 1 mg/ kg intraperitoneal injection of CNO. This, however, does not give us a precise measure of the CNO concentration within the brain., Based on literature (MacLaren et al., 2016; Mahler and Aston-Jones, 2018; Roth, 2016), a working estimate would be that CNO concentrations could be within the few hundreds of nM to tens of μM within sixty to ninety minutes post-injection based on the dose of CNO administered. It is also possible that the metabolism and pharmacodynamics of CNO may vary across strains, species, sex, and age. Our results motivate further experiments to measure brain availability of CNO within distinct regions and also across different strains, for more careful interpretation of the precise physiological effects of CNO within targeted neurocircuits. We thank the reviewers for their valuable suggestion, and we have modified our discussion section appropriately.

2. Authors should show what happens to the fEPSP amplitude when 20 μM CNO is washed out after 30-60min of continuous application. Would the amplitude recover to the previous size? This will add insights to whether CNO's effect is transient or it is altering some long-lasting cellular functions (eg. whether the effect of CNO is only desensitizing the glutamate receptors, or it is causing an effect analogous to LTD).

In order to understand whether the decline in fEPSP slope caused by administration of 20 μM CNO is transient or it can persist after the drug is removed, we carried out new experiments with slice electrophysiology wherein we washed out the CNO after 30 minutes, and recorded fEPSP for an additional 60 minutes. We first confirmed our observation that 20 μM CNO bath application evoked a significant decline in fEPSP slope. We next addressed the specific point raised by the reviewer to determine the persistence/transience of this effect evoked by the higher concentration of CNO. Interestingly, we did not observe the recovery of the decline in fEPSP slope even 60 min post-washout with aCSF, which indicates that the effect is LTD-like, and may mechanistically involve possible long-term changes. We thank the reviewers for their suggestion to perform the experiment and have now included it in our results (Extended data Fig. 2-2A, n = 7) and discussion section.

3. Authors showed, to the contrary of the commonly held view that hM3Dq's action is excitatory, there is an inhibitory effect of hM3Dq activation in a dose-dependent manner. The authors should also do whole-cell current clamp recordings and show that CNO at various doses can indeed induce a transient excitatory effect in their hands to make sure their system is working correctly as intended (eg. CNO can induce depolarizations and cause firing of CA1 pyramidal neurons early on).

As suggested by the reviewer, we have confirmed that CNO at both doses used in our manuscript 1 and 20 μM does indeed induce the predicted depolarization (Fig. 1 and Extended data Fig. 1-1). In order to measure spiking activity, neurons were held in the current-clamp mode and CA1 pyramidal cell identity was confirmed by determining the shape of the action potential. Baselines were recorded for at least five minutes. Consistent with prior literature (Alexander et al., 2009), we find that bath application of CNO can induce spiking activity both at 1 μM (Fig. 1I-J, n = 3) and at 20 μM CNO (Extended data Fig. 1-1A-B, n = 3). We appreciate the reviewer's suggestion to include this data as it strengthens the primary point of our manuscript, which is that in contrast to short-term CNO-mediated initial spiking activity, the long-term effects of hM3Dq activation by CNO in hippocampal slices can evoke distinctly different dose-dependent physiological states.

4. Fig 2: there is no change in baseline transmission with 1uM CNO; so how is there a change in PPR? If there is a change in release probability as the authors suggest, then one might expect a decrease in baseline transmission.

We observed a significant increase in paired-pulse facilitation thirty minutes following administration of 1uM CNO. While this could point to a possible potentiation of presynaptic activity, we also observe that both AMPAR and NMDAR-mediated currents decrease very early following bath application of CNO. This may explain why we do not observe a change in baseline transmission, despite observing an upregulation in PPR. In addition, we observed (1) an increase in intrinsic excitability, and (2) an altered sPSC amplitude following low dose (1 μM, 30 min) CNO treatment with a decline in cumulative probability at lower amplitudes and a shift towards increased cumulative probability at higher amplitudes (> 35 pA; Fig. 4G). We did not observe any significant change in sPSC inter-event interval (Fig. 4H) with 1 μM CNO treatment. It is also important to note that a change in sPSC may or may not arise from changes in release probability. Further, several studies in the past have suggested that although PPR is dependent on the initial release probability, they are not necessarily coupled to each other (Foster et al., 2002; Hanse and Gustafsson, 2018; Kirischuk et al., 2002; Lambert et al., 2010; Manita et al., 2007; Sun et al., 2005). Although it is unclear why we do not observe a change in baseline transmission, it is possible that the increase in paired-pulse facilitation associated pre-synaptic changes (putatively calcium-mediated), and an increase in the cumulative probability of high amplitude may compensate for the decrease in low amplitude sPSCs, and thus could potentially explain why we don't see a decline in baseline transmission.

5. Fig 2: The effects of 20 uM CNO are a little confusing; there is a depression of baseline transmission but no effect on PPR - if the depression is due to a presynaptic effect then PPR should change. Alternatively, one might speculate that the baseline depression of fEPSPs with 20uM CNO might result from the known depolarisation effects of activation of Gq coupled receptors. However, there was no reported depolarisation effects of either concentration of CNO.

Our data indicate that following administration of 20 μM CNO, there is a decline in sPSC amplitude and frequency accompanied by a decrease in AMPAR and NMDAR-mediated currents (Fig. 5). This points towards a combination of both pre-synaptic and post-synaptic mechanisms that are responsible for the downregulation of field currents as indicated by a decline in fEPSP slope. PPR in the CA1 Schaffer collaterals pathway is facilitated due to increased residual calcium that leads to enhanced release and potentiation of subsequent fEPSPs induced in close-enough intervals. Our data suggest that administration of 20 μM CNO leads to a decline in presynaptic activity, however, the non-effect observed on PPR could potentially be attributed to a floor effect. We currently lack a mechanistic understanding of why we do not see an effect on PPR. It could be potentially attributed to mechanisms other than short-term residual calcium-mediated facilitation, for example, Gq-mediated regulation of hyperpolarization activated currents, and requires further investigation.

We agree with the reviewers that the baseline depression of fEPSPs with 20uM CNO might result from the known depolarisation effects of activation of Gq coupled receptors. In experiments addressing reviewer concerns, we indeed observe depolarization followed by robust spiking activity of CA1 pyramidal neurons following both doses (1 μM and 20 μM) of CNO.

6. 1 (and 20uM) CNO lead to a decrease in AMPA EPSC amplitude; so why was there no decrease in field EPSP amplitude with 1uM CNO. In addition, given the effect of 1uM CNO on NMDA EPSCs it is surprising that LTP was still induced in the presence of 1uM CNO. The authors need to discuss why 1 μM CNO did not change the amplitude of fEPSPs, but instead reduced AMPAR and NMDAR EPSCs in whole-cell recordings.

We did not observe a decline in fEPSP slope despite a decrease in AMPA and NMDA currents following bath application of 1 μM CNO. Although we are unaware of the exact cause of this phenomenon, this might involve a compensatory potentiation of presynaptic response which was observed as an enhanced PPR in slices with 1 μM CNO administration. As indicated by reviewers despite a decline in NMDA EPSCs following 1 μM CNO, it is surprising that LTP was still induced in the presence of 1uM CNO. However, since we observe an increased postsynaptic excitability with the bath application of 1 μM CNO, it is possible that this would result in larger temporal summations during TBS which in turn can lead to more calcium influx facilitating the induction of LTP (Narayanan et al., 2010; Sehgal et al., 2013). When we induced LTP using the TBS protocol in the sham-treated controls, we only observed a significant increase in TBS-induced short-term plasticity (Fig. 3) in the 1 μM CNO-treated group as compared to sham-treated controls. However, we did not note any significant difference between the 1 μM CNO and sham-treated group in the late phase of LTP which suggests that the initial suprathreshold response following bath application of 1 μM CNO, is not persistent (55-60 min time bin; Fig. 3G). We have included the data in our results and discussion. We thank the reviewers for raising the concern.

7. Regarding the LTP experiments it was not clear what the effect of theta stimulation was in the absence of CNO, these data should be included. Next, was this a subthreshold or suprathreshold protocol for LTP under control conditions? Further, re-normalising for depression of baseline is fine, but ideally the stimulus intensity should be increased to the original baseline to control for this depression - this would be the ideal way of knowing if 20uM CNO prevents LTP per se or whether the LTP block is simply due the decrease in baseline strength.

As suggested by the reviewers, we included a sham-treated control in our experiments. We performed forty minutes of baseline recording (ten minutes baseline + thirty minutes sham for CNO treatment) following which we induced LTP using the same TBS protocol. We observed a robust LTP induction in sham-treated controls that we also noted in the 1 μM CNO-treated group, with some subtle differences. We observed a suprathreshold TBS-induced short-term plasticity in the slices that were treated with 1 μM CNO as compared to the sham-treated controls. The enhancement of short-term plasticity could be a consequence of increased PPR, high amplitude sPSC, and intrinsic excitability observed thirty minutes following administration of 1 μM CNO. We have included this result in Fig. 3. This has served as both an essential control and has provided key insights to interpret our results. We thank the reviewers for their suggestion to perform this experiment.

We agree with the reviewers that it would be interesting to know whether the failure to induce LTP is due to blockade by 20 μM CNO or simply due to a reduced baseline. However, we were unable to perform an experiment involving renormalization of depression induced by treatment of 20 μM CNO. Due to the rapid decline in AMPAR and NMDAR-mediated currents that lasts up to at least thirty minutes of CNO administration and lack of recovery of fEPSP slope following wash with aCSF (Fig. 5), we believe that the reduction in TBS-induced LTP is due to cellular changes induced by 20 μM CNO, and not simply due to a decrease in baseline.

8. Fig 5: why is there a delay in effect on NMDA EPSCs by 1uM CNO?

Activation of Gq receptors has established effects on a variety of intrinsic properties; in the initial experiments reported on P19-20 in this manuscript why are there no effects of CNO?

Some of the discussion (P26 onwards) was very speculative, especially when trying to link hM3Dq effects with mGluR and muscarinic receptor activation.

From our calcium imaging data in cultured hippocampal neurons, we observe a faster increase in Ca2+ levels following bath application of 20 μM CNO as compared to 1 μM CNO (Fig. 6). We think the delay in effect on NMDAR-mediated currents could be due to differential dynamics of calcium-mediated signaling. Further experiments are needed to understand this at a mechanistic level. We agree with the reviewers that we do not see any change in membrane properties including RMP, input resistance, sag potential, and accommodation index. Note-worthily, we observe a significant bidirectional change in intrinsic excitability following administration of the two CNO doses. Gq-mediated signaling is known to alter excitability through calcium-mediated regulation of Ih-currents (Honnuraiah and Narayanan, 2013; Neymotin et al., 2016), an inward current generated by the hyperpolarization-activated cyclic nucleotide-gated (HCN) cation channels. It is possible that other measures of intrinsic plasticity are regulated by CNO mediated Gq-activation, that have not been directly assessed in this study.

We agree that a one-on-one correlation between different Gq-coupled receptors and hM3Dq is not possible due to differences in downstream partners, distribution in different cell-types and circuit in the brain, pharmacodynamics, and doses of ligands, etc. Our aim here is to draw the attention of the readers to the fact that there are similarities between known phenotypes of endogenous Gq-coupled receptors and the synthetic hM3Dq. Also, it is essential to not take hM3Dq as a proxy for optogenetic-mediated neuronal activation using channelrhodopsin. We have tried to point this out in the discussion and we have modified our discussion to remove the speculative statements made.

Minor points:

1.Many places throughout the text and figures (eg. Line 321), “uM” should be spelled as “μM”.

We have made the necessary changes and replaced uM with μM.

2. Please explain what the circles mean in figure 1.

The circles in figures represent tetracycline transactivator (tTA) protein. We have made the necessary changes in Figure 1 and the accompanying legend explaining the same.

-

References:

Alexander, G.M., Rogan, S.C., Abbas, A.I., Armbruster, B.N., Pei, Y., Allen, J.A., Nonneman, R.J., Hartmann, J., Moy, S.S., Nicolelis, M.A., McNamara, J.O., Roth, B.L., 2009. Remote Control of Neuronal Activity in Transgenic Mice Expressing Evolved G Protein-Coupled Receptors. Neuron 63, 27-39. https://doi.org/10.1016/j.neuron.2009.06.014

Foster, K.A., Kreitzer, A.C., Regehr, W.G., 2002. Interaction of postsynaptic receptor saturation with presynaptic mechanisms produces a reliable synapse. Neuron. https://doi.org/10.1016/S0896-6273(02)01106-6

Gomez, J.L., Bonaventura, J., Lesniak, W., Mathews, W.B., Sysa-Shah, P., Rodriguez, L.A., Ellis, R.J., Richie, C.T., Harvey, B.K., Dannals, R.F., Pomper, M.G., Bonci, A., Michaelides, M., 2017. Chemogenetics revealed: DREADD occupancy and activation via converted clozapine. Science 357, 503-507. https://doi.org/10.1126/science.aan2475

Hanse, E., Gustafsson, B., 2018. Paired-Pulse Plasticity at the Single Release Site Level: An Experimental and Computational Study. J. Neurosci. https://doi.org/10.1523/jneurosci.21-21-08362.2001

Honnuraiah, S., Narayanan, R., 2013. A Calcium-Dependent Plasticity Rule for HCN Channels Maintains Activity Homeostasis and Stable Synaptic Learning. PLoS One. https://doi.org/10.1371/journal.pone.0055590

Kirischuk, S., Clements, J.D., Grantyn, R., 2002. Presynaptic and postsynaptic mechanisms underlie paired pulse depression at single GABAergic boutons in rat collicular cultures. J. Physiol. 543, 99-116. https://doi.org/10.1113/jphysiol.2002.021576

Lambert, T.J., Storm, D.R., Sullivan, J.M., 2010. MicroRNA132 Modulates Short-Term Synaptic Plasticity but Not Basal Release Probability in Hippocampal Neurons. PLoS One. https://doi.org/10.1371/journal.pone.0015182

MacLaren, D.A.A., Browne, R.W., Shaw, J.K., Krishnan Radhakrishnan, S., Khare, P., Espana, R.A., Clark, S.D., 2016. Clozapine N-Oxide Administration Produces Behavioral Effects in Long-Evans Rats: Implications for Designing DREADD Experiments. eNeuro 3, ENEURO.0219-16.2016. https://doi.org/10.1523/ENEURO.0219-16.2016

Mahler, S. V, Aston-Jones, G., 2018. CNO Evil? Considerations for the Use of DREADDs in Behavioral Neuroscience. Neuropsychopharmacology 43, 934-936. https://doi.org/10.1038/npp.2017.299

Mahler, S. V, Vazey, E.M., Beckley, J.T., Keistler, C.R., McGlinchey, E.M., Kaufling, J., Wilson, S.P., Deisseroth, K., Woodward, J.J., Aston-Jones, G., 2014. Designer receptors show role for ventral pallidum input to ventral tegmental area in cocaine seeking. Nat. Neurosci. 17, 577-85. https://doi.org/10.1038/nn.3664

Manita, S., Suzuki, T., Inoue, M., Kudo, Y., Miyakawa, H., 2007. Paired-pulse ratio of synaptically induced transporter currents at hippocampal CA1 synapses is not related to release probability. Brain Res. https://doi.org/10.1016/j.brainres.2007.03.089

Narayanan, R., Dougherty, K.J., Johnston, D., 2010. Calcium Store Depletion Induces Persistent Perisomatic Increases in the Functional Density of h Channels in Hippocampal Pyramidal Neurons. Neuron 68, 921-935. https://doi.org/10.1016/J.NEURON.2010.11.033

Neymotin, S.A., McDougal, R.A., Bulanova, A.S., Zeki, M., Lakatos, P., Terman, D., Hines, M.L., Lytton, W.W., 2016. Calcium regulation of HCN channels supports persistent activity in a multiscale model of neocortex. Neuroscience. https://doi.org/10.1016/j.neuroscience.2015.12.043

Ray, R.S., Corcoran, A.E., Brust, R.D., Kim, J.C., Richerson, G.B., Nattie, E., Dymecki, S.M., 2011. Impaired respiratory and body temperature control upon acute serotonergic neuron inhibition. Science (80-. ). https://doi.org/10.1126/science.1205295

Roth, B.L., 2016. DREADDs for Neuroscientists. Neuron 89, 683-94. https://doi.org/10.1016/j.neuron.2016.01.040

Sehgal, M., Song, C., Ehlers, V.L., Moyer, J.R., 2013. Learning to learn - Intrinsic plasticity as a metaplasticity mechanism for memory formation. Neurobiol. Learn. Mem. https://doi.org/10.1016/j.nlm.2013.07.008

Sun, H.Y., Lyons, S.A., Dobrunz, L.E., 2005. Mechanisms of target-cell specific short-term plasticity at Schaffer collateral synapses onto interneurones versus pyramidal cells in juvenile rats. J. Physiol. https://doi.org/10.1113/jphysiol.2005.093948
