# Supplementary figures and images for: Chemogenetic Activation of Excitatory Neurons Alters Hippocampal Neurotransmission in a Dose-Dependent Manner
Source: eNeuro. 2019 Nov 13;6(6):ENEURO.0124-19.2019. doi: 10.1523/ENEURO.0124-19.2019 (PMC6860986; doi:10.1523/ENEURO.0124-19.2019)

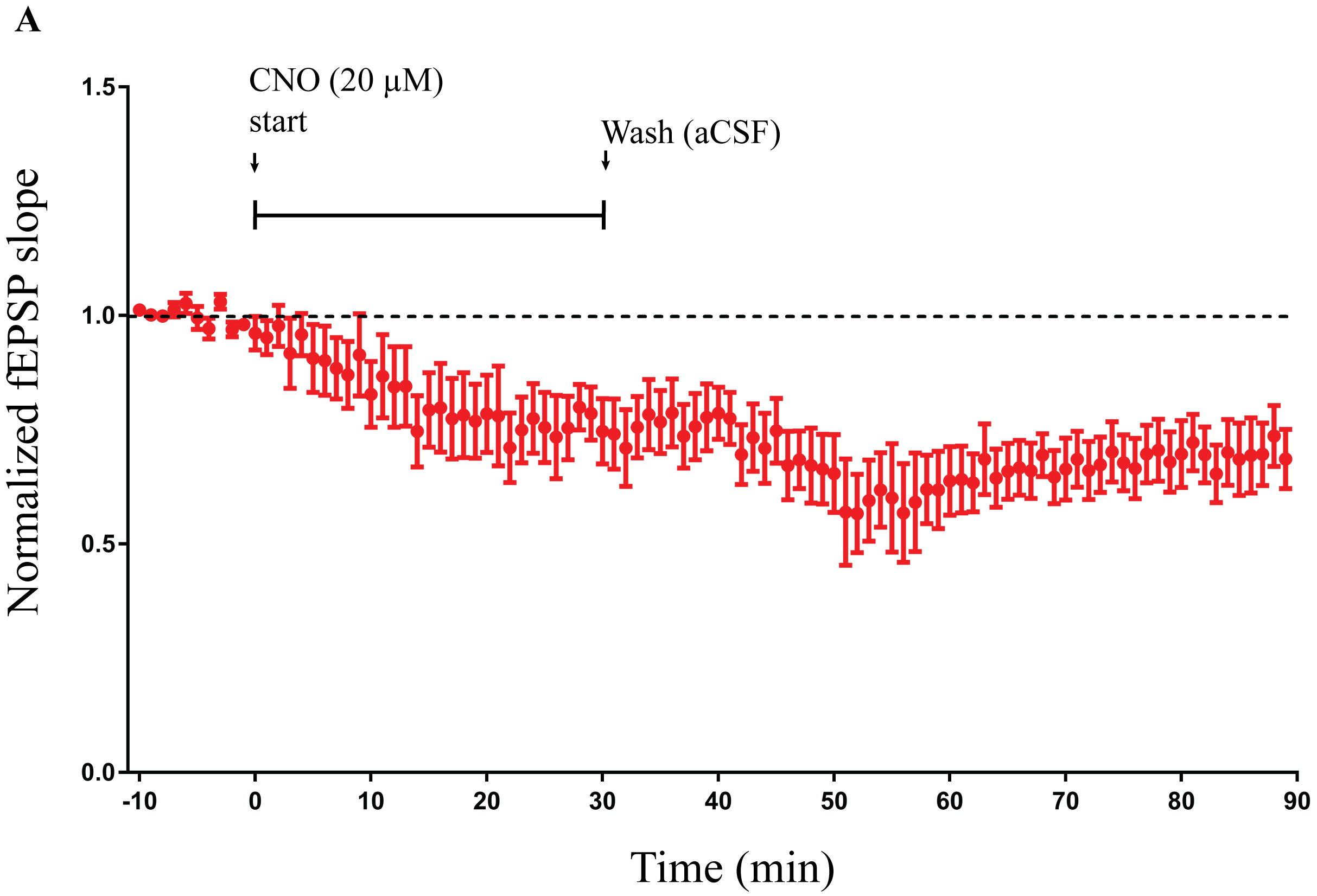

Supplement: Extended Data Figure 1-1 — A, Shown is a schematic showing whole-cell patch-clamp recording from the somata of CA1 pyramidal cells. B, Bath application of CNO (20 μM) resulted in spiking activity of CA1 pyramidal neurons. R: recording electrode. Download Figure 1-1, TIF file. [file sup_enu-eN-MNT-0124-19-s03.tif]

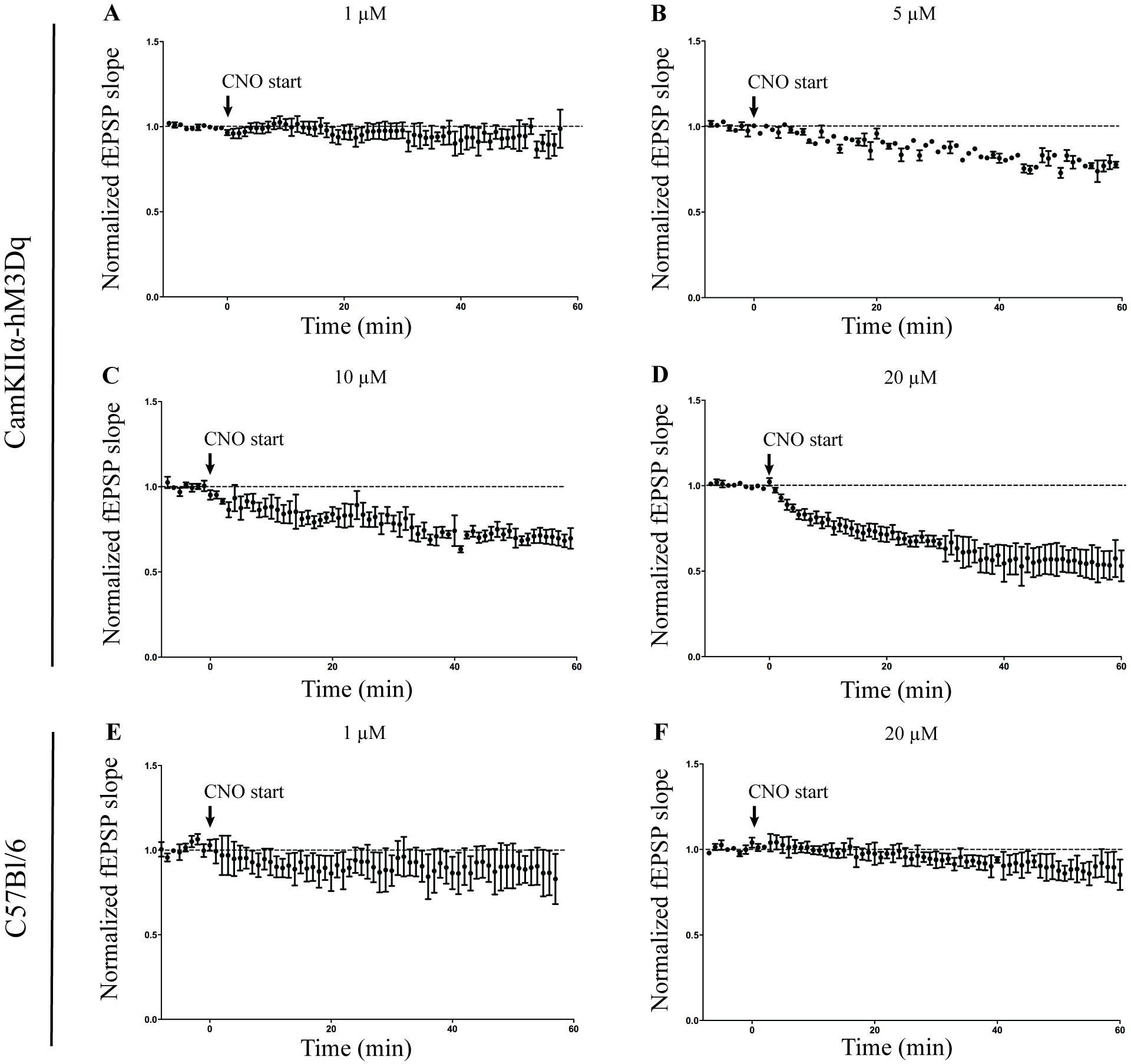

Supplement: Extended Data Figure 2-1 — Bath application of hM3Dq DREADD agonist CNO had no effect on fEPSP at low dose (1 μM; A). CNO treatment induced LTD at a subsequently high dosage of 5 μM (B), 10 μM (C), and 20 μM (D). No effect on fEPSP was observed at 1 μM (E) and 20 μM (F) CNO in slices taken from background C57Bl/6J animals. Results are expressed as the mean ± SEM. Download Figure 2-1, TIF file. [file sup_enu-eN-MNT-0124-19-s02.tif]

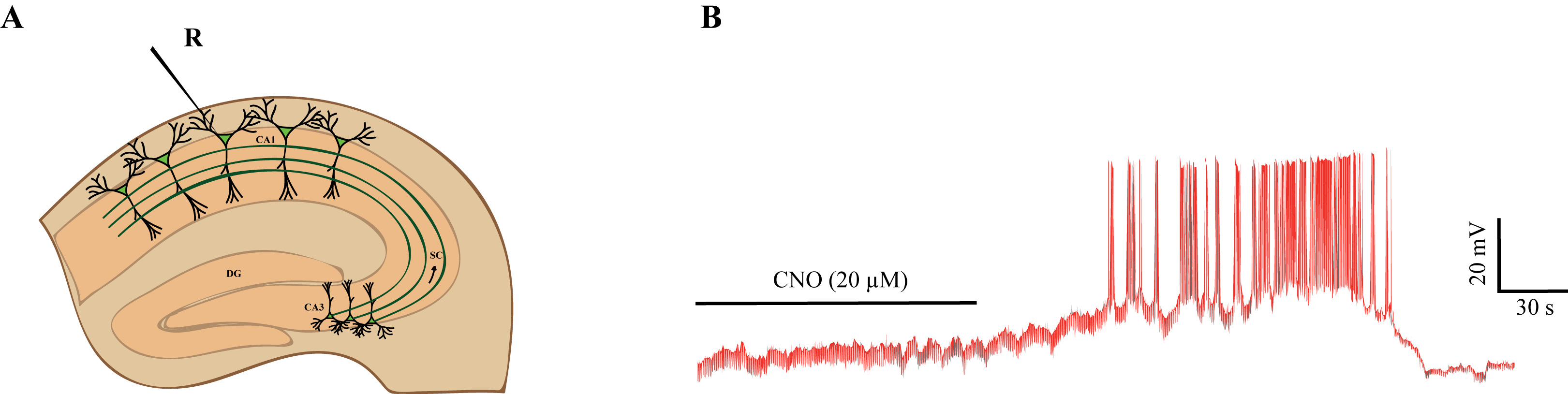

Supplement: Extended Data Figure 2-2 — A, Bath application of 20 μM CNO for 30 min resulted in decline in fEPSP slope. The fEPSP slope continued to decrease for ∼30 min after washout with aCSF before reaching a steady-state. Recovery to baseline was not observed for at least 60 min following wash with aCSF. Download Figure 2-2, TIF file. [file sup_enu-eN-MNT-0124-19-s01.tif]
